# c-MET and the immunological landscape of cancer: novel therapeutic strategies for enhanced anti-tumor immunity

**DOI:** 10.3389/fimmu.2024.1498391

**Published:** 2024-11-27

**Authors:** Parham Jabbarzadeh Kaboli, Ghazaal Roozitalab, Reyhaneh Farghadani, Zoya Eskandarian, Abdessamad Zerrouqi

**Affiliations:** 1 Department of Biochemistry, Faculty of Medicine, Medical University of Warsaw, Warsaw, Poland; 2 Noncommunicable Diseases Research Center, Fasa University of Medical Sciences, Fasa, Iran; 3 Jeffrey Cheah School of Medicine and Health Sciences, Monash University Malaysia, Subang Jaya, Selangor Darul Ehsan, Malaysia; 4 Research Institute Children’s Cancer Center, and Department of Pediatric Hematology and Oncology, University Medical Center Hamburg-Eppendorf, Hamburg, Germany

**Keywords:** c-MET, immunotherapy, PD-L1, cancer, galectin, CAR-T, antibody-drug conjugate

## Abstract

Cellular mesenchymal-epithelial transition factor (c-MET), also known as hepatocyte growth factor receptor (HGFR), is a crucial receptor tyrosine kinase implicated in various solid tumors, including lung, breast, and liver cancers. The concomitant expression of c-MET and PD-L1 in tumors, such as hepatocellular carcinoma, highlights their prognostic significance and connection to therapeutic resistance. Cancer-associated fibroblasts and mesenchymal stromal cells produce hepatocyte growth factor (HGF), activating c-MET signaling in tumor cells and myeloid-derived suppressor cells (MDSC). This activation leads to metabolic reprogramming and increased activity of enzymes like glutaminase (GLS), indoleamine 2,3-dioxygenase (IDO), and arginase 1 (ARG1), depleting essential amino acids in the tumor microenvironment that are vital for effector immune cell function. This review highlights the interplay between tumor cells and myeloid-derived suppressor cells (MDSCs) that create an immunosuppressive environment while providing targets for c-MET-focused immunotherapy. It emphasizes the clinical implications of c-MET inhibition on the behavior of immune cells such as neutrophils, macrophages, T cells, and NK cells. It explores the potential of c-MET antagonism combined with immunotherapeutic strategies to enhance cancer treatment paradigms. This review also discusses the innovative cancer immunotherapies targeting c-MET, including chimeric antigen receptor (CAR) therapies, monoclonal antibodies, and antibody-drug conjugates, while encouraging the development of a comprehensive strategy that simultaneously tackles immune evasion and enhances anti-tumor efficacy further to improve the clinical prognoses for patients with c-MET-positive malignancies. Despite the challenges and variability in efficacy across different cancer subtypes, continued research into the molecular mechanisms and the development of innovative therapeutic strategies will be crucial.

## 1 Introduction

Cellular mesenchymal-epithelial transition factor (c-MET), also known as hepatocyte growth factor receptor (HGFR), stands as a pivotal receptor tyrosine kinase (RTK) primarily situated in the epithelial cells of various tumors, encompassing those of the lung, esophagus, kidneys, breast, and gastrointestinal tract (1). Activation of c-MET predominantly occurs through interaction with its ligand, hepatocyte growth factor (HGF), secreted by fibroblasts and mesenchymal stromal cells (2, 3). The consequential binding of c-MET and HGF instigates the formation of c-MET tetramers and the phosphorylation of specific tyrosine residues, thereby initiating downstream signaling cascades. This phosphorylation event also recruits a cadre of SH2-containing proteins, including phosphatidylinositol 3,4,5-trisphosphate 5-phosphatase 2 (SHIP2), growth factor receptor-bound protein 2 (GRB2), GRB2-associated binder 1 (GAB1), and phosphatidylinositol 3-kinase (PI3K) (4). Furthermore, c-MET catalyzes the activation of mitogen-activated protein kinase (MAPK) and PI3K/Akt pathways, culminating in processes critical to cancer progression, such as cell proliferation, angiogenesis, and invasion (5).

c-MET’s intricate involvement extends beyond mere oncogenic signaling. It also plays a crucial role in bolstering the resistance of cancer cells against both targeted therapies and immunotherapies. Notably, recent attention has focused on the immunotherapeutic potential of targeting c-MET. A compendium of studies has unveiled c-MET’s ability to upregulate programmed cell death-ligand 1 (PD-L1) in diverse cancers, including liver cancer, potentially offering the tantalizing prospect of simultaneous inhibition of c-MET and PD-L1 with c-MET inhibitors (6–8). Conversely, dampening c-MET activity has been shown to stabilize PD-L1, enabling tumor cells to evade T cell responses, thereby contributing to the limited success of c-MET inhibitors in clinical trials targeting non-small cell lung cancer (NSCLC) (9). Furthermore, the development of c-MET-specific chimeric antigen receptor (CAR)-natural killer (NK) cells, as showcased in a recent 2023 study, has added an intriguing dimension to this evolving landscape (10).

This review sheds light on the complex role of c-MET in cancer pathogenesis, immune evasion, and resistance to therapeutic interventions, highlighting its pivotal role in shaping the tumor microenvironment from an anticancer immune-promoting state to a pro-tumor immune-suppressive state. Furthermore, the examination of ongoing clinical trials, the strides made in immune cell engineering, and the application of single-cell analysis all contribute to the growing significance of this research area, reinforcing its relevance and potential to push the boundaries of cancer immunotherapy.

## 2 c-MET signaling and dual inhibition of c-MET/PD-L1 pathways

**Figure 1:**
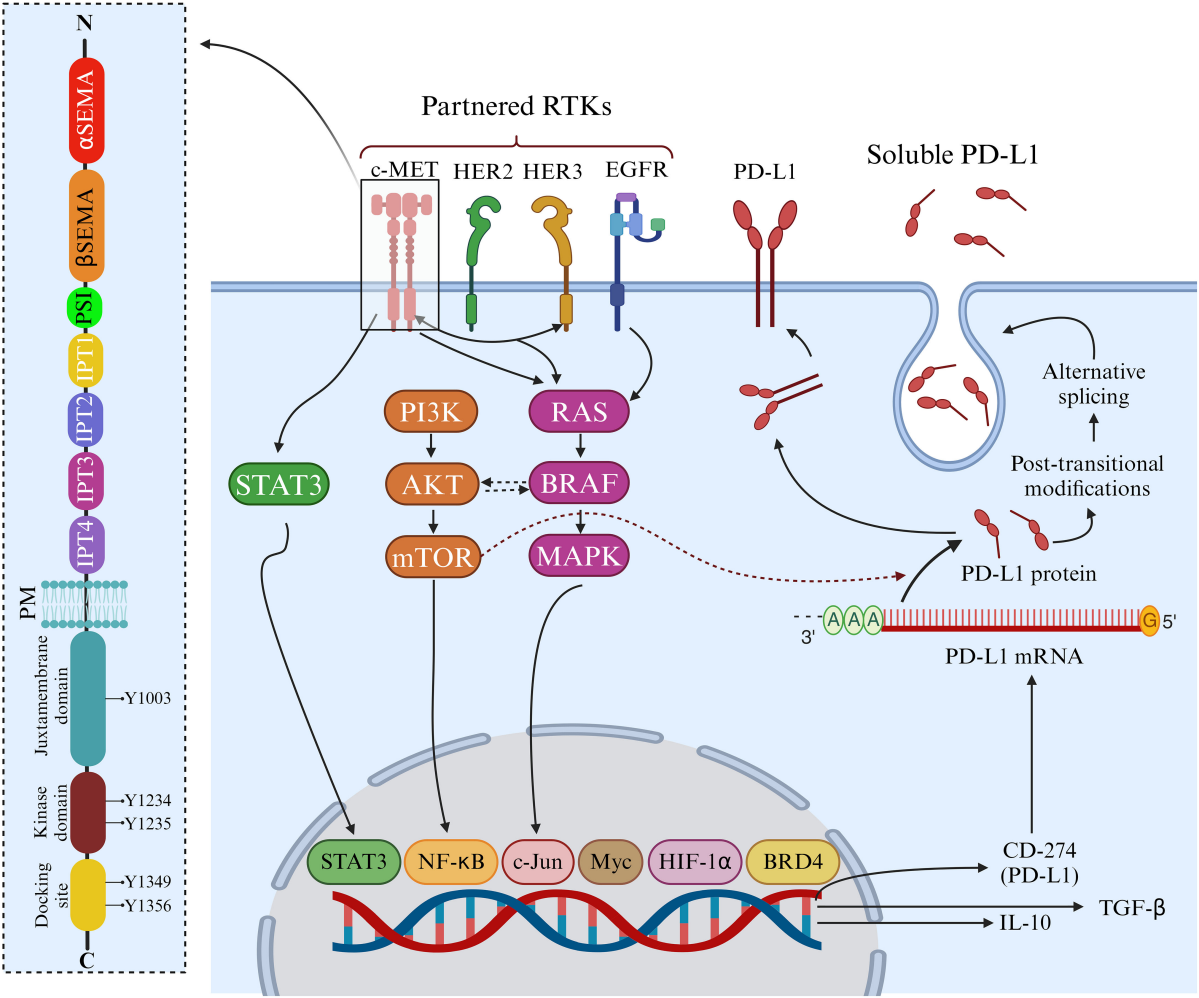
Structure and functions of c-MET. The c-MET receptor consists of α and β chains connected by three disulfide bonds. In the extracellular region, c-MET features α and β Semaphorin (SEMA) domains, a Plexin-Semaphorin-Integrin (PSI) domain, and four consecutive immunoglobulin-plexin-transcription factor (IPT1-4) domains. The intracellular region comprises a juxtamembrane domain, where phosphorylation at Y1003 regulates c-MET downregulation, a kinase domain responsible for activation through phosphorylation at Y1234 and Y1235, and a docking domain that recruits adaptor proteins and initiates c-MET signaling via phosphorylation at Y1349 and Y1356. c-MET-Mediated Upregulation of PD-L1. c-MET signaling induces the upregulation of PD-L1, IL-10, and TGF-β in specific cancers, including breast tumors, while this effect is not observed in NSCLC. This modulation is associated with regulating membrane-bound PD-L1 and soluble PD-L1, which contribute to the suppression of cytotoxic immune cells. Additionally, the serum level of soluble PD-L1 serves as a predictive marker for progressive breast cancer. *Created in BioRender. Jabbarzadeh Kaboli, P. (2024)**https://BioRender.com/m37b787*.

Situated on chromosome 7q31 in humans, the *MET* gene spans 120 kb and contains 21 exons. The production of c-MET is initiated by a 150 kDa nascent protein, which undergoes cleavage by furin endopeptidase at residues R307-S308, resulting in the formation of the mature c-MET heterodimer. This heterodimer comprises a 32 kDa extracellular α-chain and a 120 kDa transmembrane β-chain, linked by disulfide bonds. Within the extracellular portion of c-MET, several domains, including the Semaphorin (SEMA) domain, Plexin-Semaphorin-Integrin (PSI) hinge, and four immunoglobulin–plexin–transcription (IPT 1–4) domains, are present, each serving distinct functions (**Figure 1**). In the intracellular domain, c-MET harbors a juxtamembrane (JM) domain, a tyrosine kinase domain, and a crucial docking site at its c-terminus. The interaction of c-MET with HGF can be significantly impacted by mutations in c-MET, particularly in the SEMA and JM domains, which can sustain c-MET activation (5).

The intricate relationship between c-MET and PD-L1 expression is widely recognized across various tumors, and their co-expression has been implicated in conferring resistance against chemotherapy and targeted therapies (**Figure 1**) (7, 11). c-MET expression has been identified in hepatocellular carcinoma (HCC) cells resistant to the kinase inhibitor sorafenib, primarily by upregulating the MAPK pathway. In these resistant HCC cells, c-MET and PD-L1 dual expression further compound the issue, and this co-expression carries prognostic significance for HCC survival (12, 13).

Furthermore, new findings indicate that cisplatin contributes to chemoresistance by increasing HGF levels, subsequently reducing the number of CD8^+^ T cells. T cell reduction occurs through the induction of PD-L1 expression, which interacts with PD-1 (programmed cell death 1) on the membranes of T cells. Counteracting these HGF-induced effects using a c-MET inhibitor, PHA665752 has shown promise in restoring anticancer immunity (11). Similar positive correlations between c-MET and PD-L1 have been observed in renal cell carcinoma and head and neck squamous cancer cells (14, 15). Intriguingly, c-MET inhibition in NSCLC stabilizes PD-L1, resulting in resistance to PD-L1 monotherapy. This relationship suggests a negative correlation between c-MET and PD-L1 expression in NSCLC, with c-MET inhibition stabilizing PD-L1 by suppressing p-GSK-3β. This stabilization subsequently dampens T cell activity following treatment with c-MET inhibitors like tivantinib (9). Consequently, the simultaneous targeting of c-MET and PD-L1 with bispecific antibodies has emerged as an effective therapeutic strategy in c-MET/PD-L1 double-positive cancer cells, leading to promising bispecific antibodies (16, 17).

A study assessed the serum levels of HGF as a potential biomarker for forecasting clinical responses to anti-PD-1 antibody treatment. A notable correlation between c-MET activity and the PD-1/PD-L1 pathway was discovered. Higher serum concentrations of HGF, indicative of elevated c-MET activity, were associated with less favorable responses to anti-PD-1 antibody therapy. The research indicated that increased c-MET activity might suppress immune responses, as shown by the reduced secretion of perforin in CD8^+^ T cells. In contrast, inhibiting c-MET activity with a specific inhibitor like capmatinib enhanced perforin expression, thereby boosting immune activity. This suggests that c-MET inhibitors could activate the immune system and improve the effectiveness of PD-1/PD-L1-based immunotherapies, underscoring their importance in combined immunotherapy strategies (18).

On the other hand, findings suggest that high c-MET activity, reflected in elevated HGF levels, may contribute to an immunosuppressive tumor microenvironment that hampers the efficacy of PD-1/PD-L1 blockade. Therefore, co-treatment with c-MET inhibitors could potentially improve responses to cancer immunotherapy by reducing neutrophil-mediated immunosuppression and enhancing T cell activity, even in tumors that are not inherently dependent on c-MET (19). It has been found that concurrent c-MET inhibition enhances adoptive T cell transfer and checkpoint immunotherapies in murine cancer models by increasing the infiltration of effector T cells into tumors. This therapeutic benefit was independent of the tumor cells’ reliance on c-MET. Mechanistically, c-MET inhibition interfered with the reactive mobilization and recruitment of neutrophils into tumors and draining lymph nodes in response to cytotoxic immunotherapies. Without c-MET inhibition, neutrophils recruited to T cell-inflamed microenvironments rapidly developed immunosuppressive properties, thereby restricting T cell expansion and effector functions (19). Moreover, an investigation focused on how c-Met-mediated signaling, via binding to HGF, modulates apoptosis and immune escape mechanisms in renal cancer cells by regulating novel molecules heme oxygenase-1 (HO-1) and PD-L1. It was found that HGF/c-MET signaling activated the RAS/RAF pathway, down-regulated cancer cell apoptosis, and was associated with the overexpression of cytoprotective HO-1 and anti-apoptotic Bcl-2/Bcl-xL. The overexpression of HO-1 induced by c-MET was regulated at the transcriptional level—additionally, c-MET induction markedly up-regulated PD-L1 expression, which pharmacological inhibitors of c-MET could prevent. Optimal induction of PD-L1 by HGF/c-MET signaling was not achieved when either RAS or HO-1 was knocked down (20). Functional significance was studied through a co-culture assay with mouse splenocytes and murine renal cancer cells, revealing that c-MET and PD-L1 were significantly up-regulated and co-localized in human renal cancer tissues. The study suggests that c-MET promotes renal cancer cell survival by regulating HO-1 and PD-L1. This up-regulation of PD-L1 by c-MET contributes to immune escape mechanisms in renal cancer cells. The induction of PD-L1 by c-MET depends on the RAS pathway and HO-1, and inhibition of c-MET signaling or PD-L1 can increase the apoptosis of cancer cells mediated by immune cells (20).

Additionally, another study focused on the expression and function of c-MET in NK/T-cell lymphoma cells. The expression of c-MET and its ligand HGF in NK/T-cell lymphoma cell lines, nasal NK/T-cell lymphoma specimens, and patient serum samples was confirmed. HGF was shown to promote NK/T cell lymphoma proliferation in an autocrine manner. C-MET HTL epitopes are specific segments or fragments of the c-MET protein that helper T lymphocytes (HTLs) recognize. Novel c-MET HTL epitopes restricted by several HLA-DR molecules were identified. Peptide-induced helper T lines were observed to recognize and kill c-MET directly expressing NK/T-cell lymphomas and various epithelial solid tumors. c-MET-specific HTLs also recognized dendritic cells (DCs) pulsed with c-MET-expressing tumor cell lysates. It was noted that c-MET inhibition augmented HTL recognition by decreasing TGF-β production by tumor cells. These findings suggest that effective antitumor responses against c-MET-expressing tumors can be elicited by novel c-MET HTL epitopes, providing a rationale for combining c-MET targeting therapy and immunotherapy (21).

The rationale for combining c-MET inhibitors with immunotherapy is rooted in the desire to bolster the efficacy of cancer treatment by addressing multiple facets of cancer biology and immune responses. However, the clinical significance of this combination therapy can exhibit variability, contingent upon factors such as cancer subtype, pharmacological agents used, and patient demographics, all of which contribute to the heterogeneous outcomes observed in ongoing trials. These trials can reveal diverse responses depending on the markers available on different malignancies (**Table 1**). The study conducted in NSCLC illuminates the intricate interplay between c-MET inhibition and immune evasion, providing a compelling rationale for the combined treatment approach involving c-MET inhibitors and immune checkpoint blockade (9). Additionally, previous research indicates that HGF, the ligand for c-MET, exerts inhibitory effects on perforin and granzyme B, thereby suppressing antitumor immunity (22).

**Table 1:** List of clinical trials exploring the efficacy of c-MET inhibitors in combination with immunotherapeutic antibodies.

ID	Tumor(s)	Status	Phase	Started	Interventions
NCT02511184	ALK^+^NSCLC	Terminated	I	2015	Crizotinib^*^ + Pembrolizumab
NCT05782361	NSCLC	Recruiting	I	2023	Tepotinib + Pembrolizumab
NCT05700461	RCC	Not yet recruiting	I	2023	Cabozantinib^*^ + Nivolumab
NCT03149822	mRCC	Active, not recruiting	I/II	2017	Cabozantinib^*^ + Pembrolizumab
NCT03170960	Solid	Active, not recruiting	I/II	2017	Cabozantinib^*^ + Atezolizumab
NCT03468218	HNSCC	Active, not recruiting	II	2018	Cabozantinib^*^ + Pembrolizumab
NCT03534804	aUC	Recruiting	II	2018	Cabozantinib^*^ + Pembrolizumab
NCT03957551	Melanoma	Recruiting	I/II	2019	Cabozantinib^*^ + Pembrolizumab
NCT04164979	Gastric	Recruiting	II	2020	Cabozantinib^*^ + Pembrolizumab
NCT04400474	NES	Active, not recruiting	II	2020	Cabozantinib^*^ + Atezolizumab
NCT04230954	mCC	Terminated	II	2020	Cabozantinib^*^ + Pembrolizumab
NCT04139317	NSCLC	Terminated	II	2020	Capmatinib + Pembrolizumab
NCT04820179	mPanC	Recruiting	II	2021	Cabozantinib^*^ + Atezolizumab
NCT04442581	Liver	Terminated	II	2021	Cabozantinib^*^ + Pembrolizumab
NCT05007613	mESCC	Recruiting	II	2021	Cabozantinib^*^ + Atezolizumab
NCT05052723	mPanC	Recruiting	II	2022	Cabozantinib^*^ + Pembrolizumab
NCT05168618	mCRPC	Recruiting	II	2022	Cabozantinib^*^ + Atezolizumab
NCT05168163	mLiver	Recruiting	II	2022	Atezolizumab, Cabozantinib^*^, Lenvatinib
NCT05182164	aSarcomas	Recruiting	II	2022	Cabozantinib^*^ + Pembrolizumab
NCT05613413	mSNSCLC	Recruiting	II	2022	Cabozantinib^*^ + Pembrolizumab
NCT05039281	Glioblastoma	Recruiting	I/II	2022	Cabozantinib^*^ + Atezolizumab
NCT05019703	mOsteosarcoma	Recruiting	II	2023	Cabozantinib^*^ + Atezolizumab
NCT03755791	HCC	Recruiting	III	2018	Cabozantinib^*^, Sorafenib, Atezolizumab
NCT04338269	RCC	Active, not recruiting	III	2020	Cabozantinib^*^ + Atezolizumab
NCT04471428	mNSCLC	Active, not recruiting	III	2020	Cabozantinib^*^, Atezolizumab, Docetaxel
NCT04446117	mCRPC	Recruiting	III	2020	Cabozantinib^*^, Atezolizumab, Abiraterone Acetate, Enzalutamide, Prednisone

* Some c-MET inhibitors listed in this Table are multi-kinase inhibitors and can inhibit other receptor tyrosine kinases such as ALK, VEGFR, and Axl.; m, Metastatic; a, Advanced; NSCLC, Non-Small Cell Lung Cancer; RCC, Renal Cell Carcinoma; HNSCC, Head and Neck Squamous Cell Carcinoma; UC, Urothelial Carcinoma; NES, Neoplasms of the Endocrine System; CC, Cervical Cancer; PanC, Pancreatic Cancer; ESCC, Esophageal Squamous Cell Carcinoma; CRPC, Castration-Resistant Prostate Cancer; SNSCLC, Squamous Non-Small Cell Lung Cancer; HCC, Hepatocellular Carcinoma.

Consequently, emerging evidence suggests that c-MET inhibitors could activate the immune system by inhibiting c-MET and promoting perforin expression in NK and T cells. This insight underscores the potential of c-MET inhibitors to play a significant role in combination immunotherapy. Recent clinical studies exploring the synergistic potential of combining targeted c-MET therapy with immunotherapy have gained attention. Results from two terminated trials (ClinicalTrials.gov identifiers: NCT02511184 and NCT04442581) underscore the challenges of low enrollment rates, with only nine and two participants, respectively.

## 3 Advanced insights into c-MET signaling from immunological perspectives

### 3.1 Therapeutic potential of galectin family and c-MET signaling

**Figure 2:**
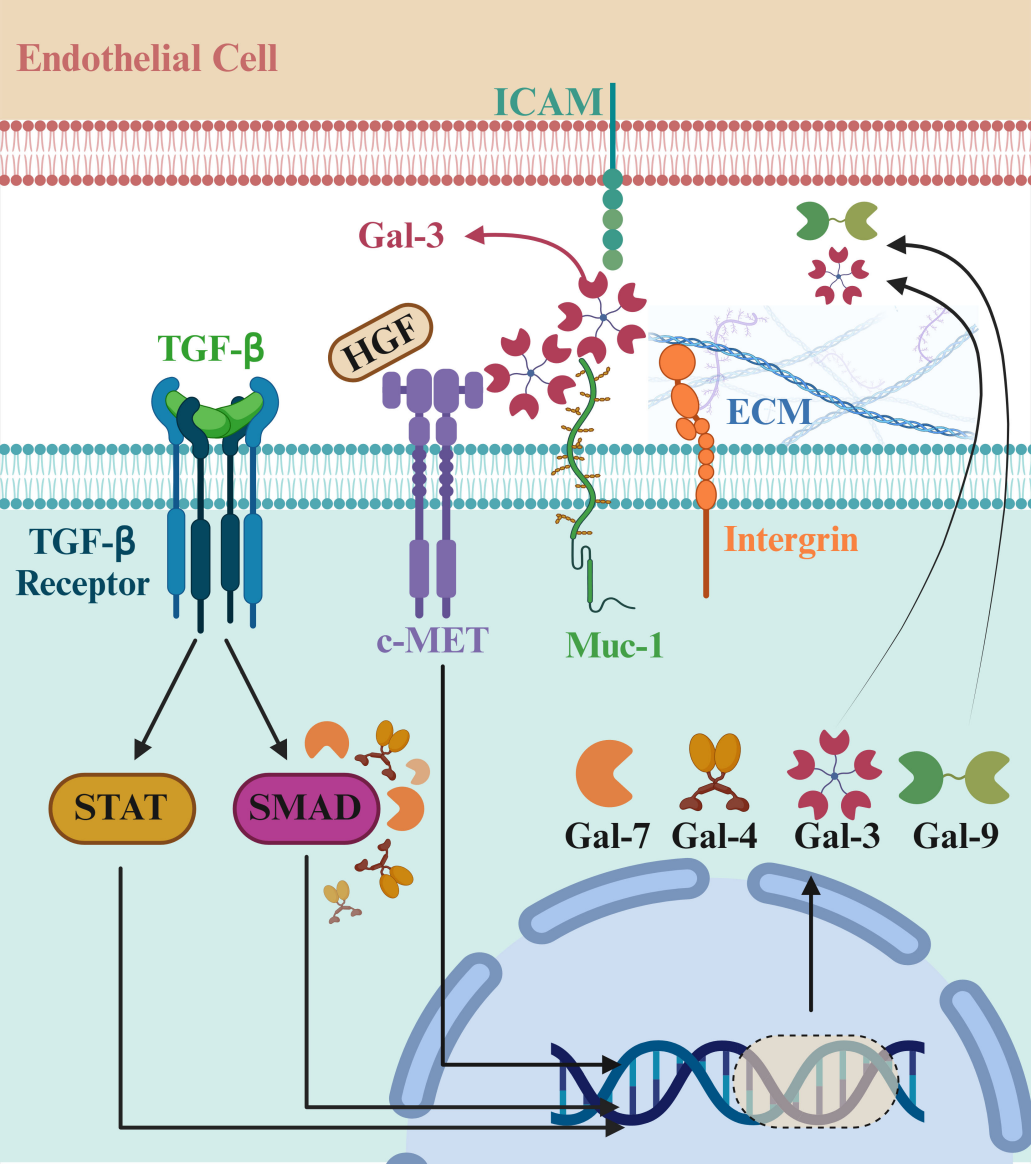
Role of galectin family members in cancer progression and c-MET signaling. The galectin family plays crucial roles in cancer biology by influencing cell adhesion, migration, and immune modulation, all critical for tumor progression. c-MET, activated by hepatocyte growth factor (HGF), interacts with galectins, indicating a connection between c-MET and Gal-3. MUC1, a cell surface glycoprotein, controls EGFR activity through Gal-3 and is linked to c-MET signaling. Additionally, galectins regulate intracellular pathways, such as the c-MET downstream protein Smad3 by Gal-7. These interactions highlight galectins as potential therapeutic targets in cancer treatment. Gal-4 and Gal-9 are dimers in which the carbohydrate recognition domain (CRD) tandemly repeats. Gal-7 is a prototype capable of forming dimers, and Gal-3 contains five units, each consisting of a CRD connected to a non-lectin part. Recently, research on the immunological role of the galectin family, especially in cancer, is emerging. So far, the upregulating effects of galectins on some receptor tyrosine kinases (RTKs) have been reported, and further research is still required to decipher the role of galectins in cancer development. *Created in BioRender. Jabbarzadeh Kaboli, P. (2024)**https://BioRender.com/t99t044*.

Galectins are a family of animal lectins that bind beta-galactosides and influence various cellular processes by interacting with cell-surface and extracellular matrix glycans. They are also present in the cytosol and nucleus, where they can impact intracellular signaling pathways through protein-protein interactions (23). Research shows galectins play crucial roles in immune and inflammatory responses, tumor development and progression, neural degeneration, atherosclerosis, diabetes, and wound repair. Studies using genetically engineered mice deficient in specific galectins have helped confirm these roles. Consequently, galectins are potential therapeutic targets or agents for treating inflammatory diseases, cancers, and other conditions (24). The intricate relationship between the galectin family and c-MET signaling underscores their pivotal roles in cancer biology. Galectins are involved in various cellular processes, including cell adhesion, migration, and immune modulation, which are critical for tumor progression (25).

c-MET and RON, two class VIII RTKs, are activated by HGF and are widely distributed on the surfaces of epithelial cells (26). Connections between class VIII RTKs and galectins are known. Proteomics research has discovered an interaction between RON and Gal-3 in human cornea-limbal epithelial cells, though the exact function of the RON-Gal-3 complex is still unclear. MUC1, a glycoprotein on cell surfaces that controls EGFR activity through its interaction with Gal-3, has also been linked to c-MET signaling, indicating that galectin family members may play a role in influencing c-MET receptor activity (27). Additionally, there is evidence of an association between MET receptor-dependent signaling and galectins inside cells, which involves the regulation of the c-MET downstream protein Smad3 by Gal-7 (**Figure 2**) (28).

Extensive research has demonstrated that galectin family members, such as Gal-7, Gal-4, and Gal-3, interact with c-MET signaling pathways to drive tumor growth, metastasis, and malignancy across different cancer types (29–31). Studies in non-melanoma skin cancer (NMSC) have shown that increased LGALS7 mRNA and Gal-7 protein expression are associated with myeloid programs, elevated CXCL1 levels, and c-MET activation. This suggests that Gal-7 is crucial in skin carcinogenesis, making it a potential therapeutic target for human NMSC (29). The upregulation of Gal-7 may contribute to the tumor microenvironment’s ability to support cancer cell survival and proliferation. By modulating immune responses and enhancing c-MET signaling pathways, Gal-7 promotes tumor growth and metastasis, indicating its critical role in the disease’s progression (29).

Similarly, in gastric cancer, Gal-4 has been identified as a critical player in peritoneal dissemination, a severe condition commonly associated with advanced stages of the disease. Gal-4 is expressed explicitly in poorly differentiated cells exhibiting a high propensity for peritoneal dissemination. Researchers employed CRISPR/Cas9-mediated genome editing to knock out the Gal-4 gene in NUGC4 cells and found that suppressing Gal-4 led to reduced cell proliferation and peritoneal metastasis (31). Further analysis revealed that Gal-4 interacts with c-MET and CD44 on the cell surface, facilitating cancer progression. The decrease in activated c-MET and CD44 levels following Gal-4 knockout indicates that Gal-4 plays a significant role in activating these pathways, promoting cancer cell proliferation and metastasis (31).

In thyroid cancer, integrating ultrasound imaging with the analysis of Gal-3, c-MET, HBME-1, and CK19 expressions has proven helpful in distinguishing between malignant and benign thyroid nodules. Immunohistochemical analysis of thyroid nodules has shown that Gal-3 and c-MET expressions are significantly higher in malignant lesions compared to benign ones. These molecular markers demonstrated a robust predictive capacity for differentiating malignant and benign nodules (30). The study found significant correlations between Gal-3 expression with nodule boundary characteristics and lymphatic metastasis and c-MET expression with both nodule micro-calcifications and lymphatic metastasis. These findings suggest elevated Gal-3 and c-MET expressions indicate more aggressive and malignant thyroid nodules. The study also highlighted that the ultrasound characteristics displayed a noteworthy ability to predict malignant nodules, with the Gal-3, c-MET, and CK19 scores increasing in alignment with higher ultrasound-assessed malignancy risk degrees (30).

Moreover, as galectins, especially Gal-9, are considered a suppressor of anticancer immunity (23), understanding the molecular mechanisms behind galectins and c-MET interactions may lead us to develop novel therapeutic agents. For instance, small molecule inhibitors or monoclonal antibodies targeting these pathways could be designed to block their interaction, thereby inhibiting tumor growth and metastasis.

Overall, the research underscores the pivotal roles of galectins—such as Gal-7, Gal-4, and Gal-3—and c-MET signaling in cancer progression. The interactions between these molecules contribute to various aspects of tumor growth, metastasis, and malignancy. By targeting these pathways, new therapeutic strategies could be developed to inhibit cancer progression and improve patient outcomes. The diagnostic and therapeutic potential of targeting the galectin family and c-MET signaling pathways offers promising avenues for more effective treatment approaches in various cancers, potentially enhancing diagnostic accuracy and patient care in clinical practice. Future research should delineate the precise molecular pathways and interactions between galectins and c-MET in different cancer types. This includes understanding how these interactions influence downstream signaling cascades, leading to tumor growth, metastasis, and immune evasion. Advanced techniques such as single-cell RNA sequencing, proteomics, and high-resolution imaging could provide deeper insights into these complex biological processes.

The intricate interplay between the galectin family and c-MET signaling represents a significant frontier in cancer research and therapy. The growing body of evidence underscores the critical roles of galectins like Gal-7, Gal-4, and Gal-3, as well as c-MET, in tumor growth, metastasis, and malignancy. By targeting these pathways, novel therapeutic strategies can be developed to inhibit cancer progression and improve patient outcomes effectively. Integrating molecular marker analysis with advanced imaging techniques, personalized medicine, and combination therapies holds great promise for enhancing diagnostic accuracy and patient care in clinical practice. Continuing research and clinical innovation will be essential to fully realize the potential of galectin and c-MET-targeted therapies in the fight against cancer.

### 3.2 Crosstalk between c-MET and TGF-β

Transforming growth factor (TGF)-β, a multifunctional cytokine, plays a pivotal role in cancer. In early tumorigenesis, it acts as a tumor suppressor, inhibiting cell proliferation and inducing apoptosis. However, in advanced stages, cancer cells often exploit TGF-β signaling to promote tumor progression and metastasis. TGF-β facilitates epithelial-mesenchymal transition (EMT), enhances cell invasiveness, and modulates the TME to support immune evasion. Notably, the potential of TGF-β as a therapeutic target in cancer progression is a crucial focus of this research. Furthermore, emerging evidence suggests a significant crosstalk between c-MET and TGF-β pathways contributing to immune evasion in cancer (32).

The role of TGF-β in inducing epithelial-mesenchymal transition and regulating various cellular processes is well-documented. Dysregulation of TGF-β signaling is frequently associated with cancer progression and poor clinical outcomes. Fangchinoline (FCN) has been investigated for its potential to inhibit TGF-β-induced EMT in colon cancer cells. FCN treatment suppressed TGF-β-induced mesenchymal markers and enhanced epithelial markers at both protein and mRNA levels. Additionally, FCN was found to inhibit the activation of c-MET/PI3K/Akt/mTOR signaling pathways, which are crucial for cell proliferation and migration. These findings suggest that FCN, which inhibits c-MET and TGF-β, may offer therapeutic benefits by modulating key signaling pathways in cancer progression (33).

On the other hand, pancreatic cancer (PC), characterized by its aggressive nature and poor prognosis, has also been a focus of c-MET pathway research. Pancreatic stellate cells (PSCs), which interact with cancer cells, drive tumor growth and metastasis through the HGF/c-MET pathway. A novel therapeutic strategy combining HGF/c-MET pathway inhibition with gemcitabine chemotherapy demonstrated promising results. This combination reduced tumor volume, suppressed EMT and stemness characteristics, and eradicated metastatic lesions. Additionally, the therapy decreased TGF-β secretion by cancer cells, promoting cytotoxic T cell infiltration and enhancing cancer cell death. These findings provide a strong rationale for clinical trials to evaluate the efficacy of this combined approach in managing pancreatic cancer (34).

Genomic MET amplification and exon 14 skipping are currently established as clinically significant biomarkers used to stratify subsets of NSCLC patients based on their predicted response to c-MET inhibitors. While these biomarkers have provided valuable insights, the overall clinical efficacy of this stratification strategy remains somewhat limited. Notably, c-MET protein overexpression, a common occurrence in approximately 20-25% of NSCLC patients, has yet to be definitively characterized as a clinically actionable biomarker. Consequently, there is a clear need for an optimized approach to accurately categorize patients with c-MET overexpression for informed decision-making regarding c-MET inhibitor treatment.

Recent research has shed light on the role of SYK in regulating cellular plasticity and its association with sensitivity to c-MET inhibitors, both *in vitro* and *in vivo*, particularly in patient-derived xenograft (PDX) models exhibiting c-MET overexpression, regardless of MET gene status (35). Treatment with TGF-β1 leads to the downregulation of SYK transcription, increased Sp1-mediated transcription of FRA1, and the restoration of a mesenchymal state in cells, consequently conferring resistance to c-MET inhibitors. Importantly, clinical studies have shown that a subset of NSCLC patients with concomitant c-MET and SYK overexpression demonstrated a notably high response rate of 73.3% and longer PFS when treated with c-MET inhibitors compared to other patient subgroups. Conversely, combining SYK negativity and TGF-β1 positivity was associated with *de novo* and acquired resistance to c-MET inhibitors. These findings suggest that SYK modulates cellular plasticity toward a therapy-sensitive epithelial state (35).

Emerging evidence suggests a significant crosstalk between c-MET and TGF-β pathways that contributes to immune evasion in cancer. This interaction occurs at multiple levels, influencing cancer cells’ intrinsic properties and the immune microenvironment’s extrinsic modulation. TGF-β-induced EMT enhances cancer cell invasiveness and metastatic potential, with c-MET signaling synergizing to promote EMT and stem-like properties. This cooperation supports tumor cell dissemination and resistance to immune-mediated cytotoxicity. EMT-associated changes, such as the downregulation of epithelial markers and upregulation of mesenchymal markers, reduce recognition and elimination by immune cells (36).

Both c-MET and TGF-β independently and cooperatively suppress anti-tumor immunity. TGF-β inhibits the activation and function of various immune cells (37, 38). In contrast, c-MET activation recruits Tregs and induces the expression of immune checkpoint molecules, such as PD-L1, on tumor cells (39). The TME is remodeled by c-MET and TGF-β signaling pathways. c-MET-driven HGF secretion recruits myeloid-derived suppressor cells (MDSCs) and tumor-associated macrophages (TAMs), while TGF-β induces fibroblasts to differentiate into cancer-associated fibroblasts (CAFs), creating a barrier against immune cell infiltration (40).

The interplay between c-MET and TGF-β in immune evasion underscores the potential of combinatorial therapeutic strategies targeting both pathways (41). c-MET inhibitors, such as crizotinib, cabozantinib, and tivantinib, have shown promise in inhibiting tumor growth and metastasis but can be limited by compensatory mechanisms and immunosuppressive TME. Combining c-MET inhibitors with TGF-β pathway antagonists offers a promising approach to enhance anti-tumor immunity (34). TGF-β pathway (JAK/STAT) inhibitors, including small molecules, neutralizing antibodies, and ligand traps, disrupt TGF-β signaling and its immunosuppressive effects (42). Preclinical studies demonstrate that dual inhibition of c-MET and TGF-β pathways synergistically reduces tumor growth, metastasis, and immune evasion, highlighting the potential of this approach (43).

Several clinical trials investigate the efficacy of combining c-MET inhibitors with TGF-β antagonists or immune checkpoint inhibitors. Combining c-MET inhibitors with PD-1/PD-L1 inhibitors is being evaluated for its potential to enhance the anti-tumor immune response (44).

Understanding the molecular mechanisms underlying c-MET and TGF-β crosstalk can inform the development of novel biomarkers for patient stratification and treatment optimization. Biomarkers such as c-MET and TGF-β expression levels, EMT transcription factors, and immune cell infiltration profiles could help identify patients most likely to benefit from combination therapies, enabling more personalized and effective treatment strategies.

Despite the promising potential of targeting c-MET and TGF-β pathways, several challenges need addressing. The redundancy and complexity of signaling networks in cancer cells can lead to adaptive resistance mechanisms, necessitating the identification and targeting of additional compensatory pathways. The heterogeneous nature of tumors and the dynamic TME require a comprehensive understanding of the temporal and spatial aspects of c-MET and TGF-β signaling.

**Figure 3:**
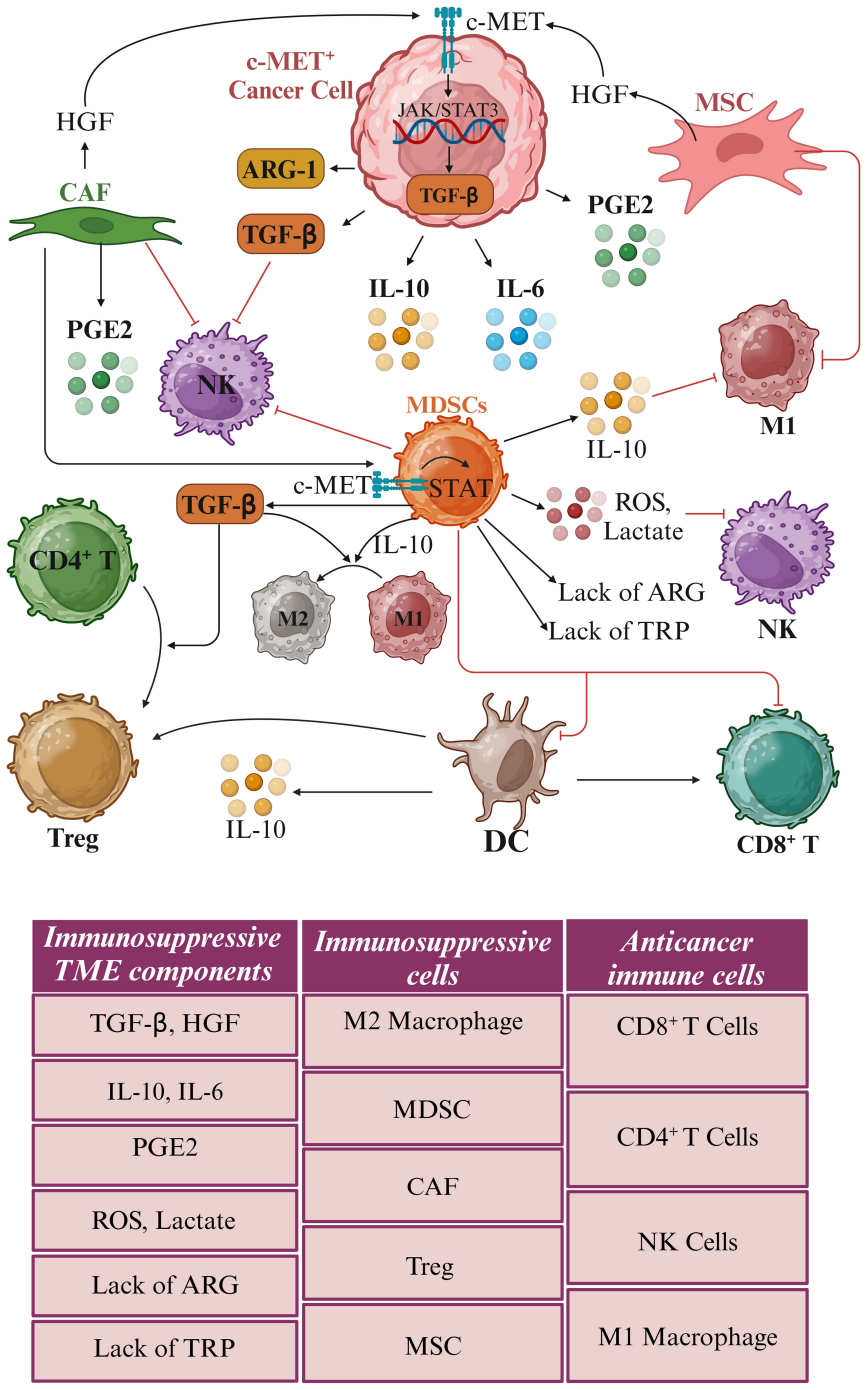
Impact of c-MET amplification on immune cell functionality. Hepatocyte growth factor (HGF) released by cancer-associated fibroblasts (CAFs) and mesenchymal stromal cells (MSCs) triggers the c-MET receptors predominantly found on c-MET^+^ cancer cells and myeloid-derived suppressor cells (MDSCs). The amplification of c-MET leads to heightened secretion of transforming growth factor-beta (TGF-β), a cytokine crucial for immune modulation. Elevated TGF-β levels drive the polarization of M1 macrophages, typically pro-inflammatory and anti-tumorigenic, toward M2 macrophages, characterized by immunosuppressive and pro-tumorigenic properties. Furthermore, TGF-β prompts converting CD4^+^ T helper cells into regulatory T cells (Tregs), recognized for their immune-suppressive role. This immunosuppressive milieu, orchestrated by M2 macrophages and Tregs, effectively dampens the body’s anti-cancer immune responses, enabling tumors to evade immune surveillance and destruction. The hypoxic tumor microenvironment (TME) is characterized by elevated levels of reactive oxygen species (ROS), lactate, and cytokines such as TGF-β. It also features a deficiency of the amino acids arginine (ARG) and tryptophan (TRP), along with an increased presence of myeloid-derived suppressor cells (MDSCs), M2 macrophages, regulatory T cells (Tregs), cancer-associated fibroblasts (CAFs), and mesenchymal stem cells (MSCs). These conditions create an environment that facilitates tumor immune evasion. This distinct immune landscape emphasizes the crucial role of c-MET expression levels in shaping the TME and impacting the immune system’s ability to identify and eradicate cancer cells. *Created in BioRender. Jabbarzadeh Kaboli, P. (2024)**https://BioRender.com/i50x671*.

The relationship between c-MET and TGF-β in cancer cell immune evasion represents a compelling area of research with significant therapeutic implications (**Figure 3**). The crosstalk between these pathways contributes to multiple aspects of tumor progression, including EMT, immune suppression, and TME modulation. Targeting both c-MET and TGF-β pathways holds promise for enhancing the efficacy of cancer therapies and overcoming immune evasion. Ongoing clinical trials and preclinical studies are shedding light on the potential benefits of combinatorial approaches, paving the way for more effective and personalized treatments.

### 3.3 c-MET-targeted therapy: insights from immune single-cell analysis

First and foremost, the use of single-cell analysis has shed light on the pivotal role of HGF/c-MET signaling in the crosstalk between two distinct subgroups of liver endothelial cells: HGF-expressing liver sinusoidal endothelial cells (LSEC) and c-MET-expressing continuous endothelial cells (CEC). This intricate interaction ultimately drives the replacement of LSEC with CEC in liver carcinogenesis. Notably, c-MET signaling has initiated the transition from LSEC to CEC (45). Moreover, single-cell analysis of circulating tumor cells has unveiled compelling insights into the effectiveness of HGF/c-MET inhibitors, such as rilotumumab (formerly AMG102) and compound A (a c-MET small molecule inhibitor), in curtailing cancer progression post-surgery. This revelation prompted an investigation into circulating pancreatic stellate cells and the role of anti-c-MET adjuvant therapy in a newly developed mouse model following pancreatic tumor resection. The study revealed that HGF/c-MET dual inhibition curtailed angiogenesis and reduced the number of circulating pancreatic tumor cells (31).

In parallel, single-cell transcriptome analysis has brought to the forefront the critical functionality of c-MET signaling within glioblastoma cells navigating the hypoxic TME. Targeting c-MET with inhibitors like cabozantinib, crizotinib, foretinib, PF04217903, and tivantinib disrupted antioxidant defenses, culminating in apoptotic cancer cell death. Furthermore, the synergy between c-MET inhibitors and the DNA-alkylating drug temozolomide was explored, revealing promising synergistic effects (46, 47). Despite the limited scope of studies on c-MET signaling through single-cell analysis, both bulk and single-cell analyses converge to emphasize the overexpression of c-MET within a distinct subpopulation of glioma cells that exhibit traits such as strong hypoxia, inflammation, stem-like properties, metastatic potential, and neoplastic characteristics among ten subpopulations (46).

Transitioning to a different facet of the TME, TAMs emerge as a pivotal component. These TAMs can be classified into two primary phenotypes: M1 TAMs, known for their pro-inflammatory and anti-tumor attributes, and M2 TAMs, characterized by their anti-inflammatory and pro-tumor properties (48). Within this context, RNA-sequencing analysis in gastric cancer uncovered a link between macrophage-derived IL-10 and the activation of c-MET/STAT3 signaling pathways. IL-10 was found to be significantly elevated in both gastric tumor tissues and the serum of afflicted individuals, with TAMs identified as the primary source. Further exploration revealed that IL-10 may trigger the activation of the c-MET/STAT3 signaling pathway, fueling gastric cancer progression. These findings underscore the potential of IL-10 as a promising therapeutic target in gastric cancer treatment (49).

c-MET signaling also significantly influenced TAMs-specific cytokines (**Figure 3**). Markers such as IL-10 and TGF-β were associated with pro-regenerative M2 macrophages, while iNOS, TNFα, IL-1, IL-12, IL-18, and IFN-γ indicated pro-inflammatory M1 macrophages (50). Notably, HGF/c-MET signaling emerged as a trigger for the activation of PI3K/Akt signaling and concurrent inhibition of NF-κB signaling in M1 macrophages, leading to the release of IL-10 and TGF-β. Intriguingly, the expression of iNOS, TNF-α, and IL-6 in these macrophages decreased following treatment with HGF, suggesting that c-MET signaling induces an M1-to-M2 transition in TAMs (51). Conversely, the inhibition of HGF/c-MET signaling through PHA-665752 reversed these effects, promoting the maintenance of M1 macrophages by elevating IL-1β and iNOS (52).

**Figure 4:**
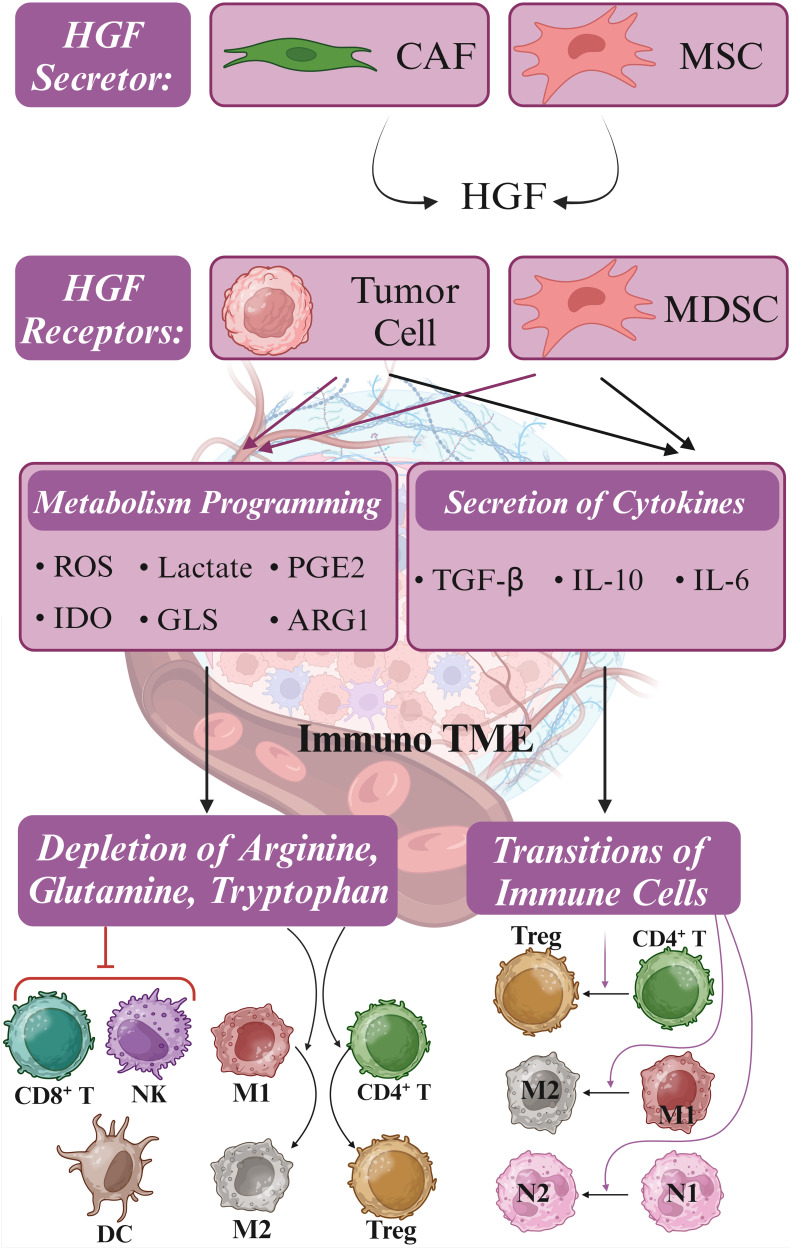
Metabolism reprogramming and immune suppressive TME. Hepatocyte growth factor (HGF) is secreted by cancer-associated fibroblasts (CAFs) and mesenchymal stromal cells (MSCs), stimulating c-MET signaling in tumor cells and myeloid-derived suppressor cells (MDSCs). Upon activation of c-MET, metabolic reprogramming is initiated, resulting in the release of Indoleamine 2,3-dioxygenase (IDO), glutaminase (GLS), and arginase 1 (ARG1). These metabolic changes result in the depletion of CD8^+^ T cells, NK cells, and dendritic cells (DCs) by limiting essential amino acids such as arginine, glutamine, and tryptophan required for immune cell function. Concurrently, tumor cells and MDSCs release transforming growth factor-beta (TGF-β) and interleukin-10 (IL-10), inducing the polarization of neutrophils, macrophages, and T cells from anti-tumor (M1, N1, and CD4^+^) to pro-tumor (M2, N2, and Treg) phenotypes. This intricate interplay between tumor cells and MDSCs presents multiple molecular targets for combating immune evasion mechanisms. Furthermore, targeting GLS, IDO, and ARG1, along with modulating the JAK/STAT pathway and employing checkpoint inhibitors, may synergize with c-MET inhibitors in enhancing the efficacy of c-MET-targeted immunotherapy. *Created in BioRender. Jabbarzadeh Kaboli, P. (2024)**https://BioRender.com/l42g986*.

Transitioning to another facet of the tumor microenvironment, Tregs play a critical role in inhibiting various immune cells, including CD8^+^ and CD4^+^ T cells, dendritic cells, and NK cells. Targeting crucial markers like CD25, forkhead box p3 (FoxP3), TGF-β receptor, and indoleamine 2,3-dioxygenase 1 (IDO-1), arginase 1 (ARG1), and glutaminase (GLS) holds promise in inducing antitumor immunity (**Figure 4**) (32, 53). In colorectal cancer liver metastasis, the emergence of CD4^+^FOXP3^+^ Tregs, coupled with elevated levels of α-smooth muscle Actin, HGF, and c-MET, suggests potential therapeutic targets for this metastatic variant of colorectal cancer (54). Furthermore, in gastric cancer, HGF and c-MET were implicated in Treg accumulation in peripheral blood. The expression of c-MET in circulating monocytes of gastric cancer patients was identified, with monocyte-derived dendritic cells exposed to HGF exhibiting a regulatory phenotype. Interestingly, treatment with an anti-HGF antibody reduced circulating Tregs among gastric cancer patients, highlighting the role of HGF in fostering Treg accumulation indirectly via c-MET-expressing monocytes. These findings suggest the potential benefits of HGF/c-MET targeted therapies, including combinations with immune checkpoint inhibitors, in cancer treatment (41).

Shifting the focus to immunogenic cell death (ICD) in breast cancer, limitations associated with chemotherapy-induced ICD are highlighted. A novel approach employing crizotinib polymerized prodrug carriers to enhance ICD, combined with doxorubicin, demonstrated extended circulation, efficient tumor accumulation, and simultaneous drug release within tumor cells. This combination resulted in synergistic ICD induction, enhanced cytotoxic T lymphocyte infiltration, reduced Treg cells, increased cytokine secretion, and ultimately improved antitumor activity in a breast cancer mouse model. This innovative nano-drug delivery system holds promise for enhancing the efficacy of chemo-immunotherapy in breast cancer (55).

Tumor-associated neutrophils (TANs) constitute another critical element recruited to the cancer microenvironment. N1 TANs exert antitumor effects, while N2 TANs promote immune suppression, tumor progression, angiogenesis, and metastasis. Elevated TAN abundance or neutrophil-to-lymphocyte ratio in tumor patients correlates with unfavorable prognoses (56). TGF-β plays a significant role in neutrophil plasticity, guiding these immune cells toward acquiring the N2 phenotype. This process involves complex signaling pathways and interactions that ultimately result in neutrophil’s functional and phenotypic transformation, highlighting the importance of TGF-β in modulating immune responses and cellular behavior (**Figure 4**) (57, 58). The finding that IL-10 reduces the synthesis of pro-inflammatory cytokines such as IL-1β and TNF-α in neutrophils provides insights into the polarized functional states of these immune cells (59, 60).

Multiple studies have shown that c-MET signaling modulates immune cell composition in the tumor microenvironment, particularly immunosuppressive neutrophils (61, 62). Research focusing on metastatic castration-resistant prostate cancer revealed that c-MET is essential for recruiting and activating neutrophils in response to HGF. Inhibiting c-MET resulted in heightened tumor growth and metastasis, underscoring the pivotal role of c-MET in neutrophil infiltration. Furthermore, systemic treatment with c-MET inhibitors curtailed the recruitment of TANs, ultimately impacting tumor growth and volume more significantly than *MET* knockdown in cancer cells (19).

Conversely, another study uncovered that c-MET inhibition could enhance immunotherapy’s effectiveness by promoting neutrophil infiltration. Inhibiting c-MET using potent c-MET inhibitors capmatinib and PF-04217903 alongside anti-PD-1 checkpoint immunotherapy increased infiltrating neutrophils and heightened immunotherapy effectiveness. When wild-type neutrophils were exposed to HGF, it enhanced their ability to adhere to and migrate across an activated endothelial layer. In contrast, neutrophils lacking the c-MET knockout did not exhibit this increased adhesion and chemotaxis in response to HGF stimulation, suggesting that the c-MET plays a crucial role in mediating the effects of HGF on neutrophil adhesion and migration through activated endothelium (63). The combination of c-MET inhibition and immunotherapy also augmented the count and functionality of antigen-specific CD8^+^ T cells within tumor tissues, highlighting its potential impact on the tumor microenvironment. Nonetheless, factors like IL-6, TGF-β, and IL-10, secreted by tumor cells, perpetuate chronic inflammation, stimulating immunosuppressive myeloid-derived suppressor cells, macrophages, and neutrophils. This, in turn, sustains an inflammatory environment where IL-10 from neutrophils inhibits the antitumor properties of M1 TAMs while promoting the activity of tumorigenic M2 TAMs (**Figure 4**) (57).

Recent advancements in understanding the complex interplay between the tumor microenvironment, immune cells, and targeted therapies have opened new avenues for improving cancer treatment outcomes. The role of TANs in cancer progression and immune suppression has also gained significant attention. The plasticity of neutrophils, influenced by factors such as TGF-β and IL-10, highlights the importance of understanding the molecular mechanisms governing their functional polarization. The c-MET signaling pathway has emerged as a crucial player in regulating immune cell composition in the tumor microenvironment, particularly immunosuppressive neutrophils, macrophages, and Treg. Despite the mixed results of c-MET inhibition, with some studies demonstrating enhanced tumor growth and metastasis while others suggest improved immunotherapy efficacy, its potential cannot be overlooked. The ability of c-MET inhibition to promote neutrophil infiltration and improve the functionality of antigen-specific CD8^+^ T cells highlight its potential as a complementary approach to immunotherapy. Tumor cells secreting factors such as IL-6, TGF-β, and IL-10 perpetuate an inflammatory environment that supports tumor growth and suppresses antitumor immune responses (**Figure 4**).

In conclusion, the dynamic interactions between cancer cells, immune cells, and the tumor microenvironment present challenges and opportunities for developing effective cancer therapies. Targeted approaches that modulate neutrophil function enhance ICD and harness the immune system’s power hold promise for improving patient outcomes. However, the urgent need for further research to unravel the complex signaling pathways and molecular mechanisms that govern these interactions is undeniable, enabling the development of more precise and personalized cancer treatment strategies.

## 4 Anti-MET antibody therapies

Inhibitors of the HGF/c-MET signaling pathway fall into small-molecule compounds and biologics. Small-molecule compounds block the pathway by inhibiting the tyrosine kinase activity and autophosphorylation of c-MET, often affecting multiple RTKs. On the other hand, biologics—including truncated HGF, the N-terminal SEMA domain of HGF, the soluble extracellular domain of c-MET (decoy MET), and antibodies targeting HGF and c-MET—suppress the pathway by preventing the interaction between HGF and c-MET. Biologics typically offer more specific inhibition of the HGF/c-MET signaling pathway than small molecules. Several therapeutic antibodies targeting this pathway are currently under preclinical and clinical development (5, 64).

### 4.1 Anti-MET/HGF monoclonal antibodies

#### 4.1.1 Rilotumumab (AMG102, Amgen)

Rilotumumab is a human monoclonal antibody designed to target HGF, preventing its interaction with the c-MET receptor and inhibiting the related cellular processes and in a Phase II clinical trial for gastric and esophagogastric junction cancers, rilotumumab combined with epirubicin, cisplatin, and capecitabine (ECX) demonstrated improvements in both progression-free survival (PFS) and overall survival (OS) for patients with high c-MET expression. Patients were administered either a placebo or rilotumumab (15 mg/kg or 7.5 mg/kg) alongside ECX every three weeks. The median PFS was 5.1 months for the 15 mg/kg dose, 6.8 months for the 7.5 mg/kg dose, and 4.2 months for the placebo. Objective response rates (ORRs) were 31% for the 15 mg/kg dose, 48% for the 7.5 mg/kg dose, and 21% for the placebo. Median OS was 9.7 months for the 15 mg/kg group, 11.1 months for the 7.5 mg/kg group, and 8.9 months for the placebo group. Adverse events, such as hematologic issues, peripheral edema, and venous thromboembolism, were more frequent in the rilotumumab groups (65). These favorable outcomes have prompted a Phase III study (RILOMET-1) in c-MET^+^ gastric and gastroesophageal junction cancers.

Moreover, rilotumumab has proven effective in metastatic colorectal cancer with wild-type KRAS, where a Phase II trial found a median PFS of 5.2 months for the combination of rilotumumab and panitumumab, compared to 3.7 months for panitumumab alone (ClinicalTrials.gov identifier: NCT00719550) (66). In addition, the potential pharmacokinetic (PK) drug-drug interactions (DDI) between rilotumumab and the chemotherapy regimen of epirubicin, cisplatin, and ECX were also evaluated. In this Phase III double-blinded, placebo-controlled study, rilotumumab, epirubicin, and cisplatin were administered intravenously every three weeks, while capecitabine was taken orally twice daily. PK samples for rilotumumab were collected pre-dose and post-infusion during multiple cycles, and ECX-PK samples were collected during cycle 3. Non-compartmental analyses were used to compare PK parameters between the treatment arms, and a population PK model from previous studies was employed to predict rilotumumab serum concentrations. Additionally, observed rilotumumab serum concentrations matched model predictions, indicating no impact on rilotumumab exposure by ECX. These results suggest a lack of PK-based DDI between rilotumumab and ECX (ClinicalTrials.gov identifier: NCT00719550) (67).

#### 4.1.2 Ficlatuzumab (AV-299; SCH 900105, AVEO Pharmaceuticals)

Ficlatuzumab, developed by AVEO Pharmaceuticals under the names AV-299 and SCH 900105, is a humanized monoclonal antibody designed to target HGF, thus disrupting the HGF-induced c-MET signaling pathway by interfering with the interaction between HGF and c-MET (68). Studies conducted using the H596 NSCLC xenograft model have indicated that combining ficlatuzumab with epidermal growth factor receptor (EGFR) inhibitors such as erlotinib or cetuximab enhances its anti-cancer effects compared to using either agent alone. In a Phase I trial involving patients with various advanced solid tumors, including sarcoma, ovarian cancer, mesothelioma, and glioblastoma multiforme, ficlatuzumab was administered intravenously at varying doses biweekly (69). In subsequent Phase II trials, ficlatuzumab was administered alongside daily doses of erlotinib at the recommended Phase II dosage. Adverse events reported during ficlatuzumab monotherapy included fatigue, peripheral edema, headache, hematologic complications, and pruritus, while combination therapy commonly resulted in rash and diarrhea. The Phase I trial confirmed the safety and tolerability of ficlatuzumab when used alongside erlotinib, leading to a subsequent Phase II study evaluating the efficacy of gefitinib alone or in combination with ficlatuzumab in NSCLC patients (70).

Additionally, a multicenter, randomized phase II study evaluated the efficacy of ficlatuzumab, a monoclonal antibody targeting hepatocyte growth factor, when administered alone or combined with cetuximab in patients diagnosed with recurrent or metastatic head and neck squamous cell carcinoma (HNSCC) who had previously shown resistance to cetuximab. Activation of the hepatocyte growth factor/c-MET pathway is recognized as a resistance mechanism to cetuximab in HNSCC. The study aimed to assess the median PFS. The trial between 2018 and 2020 randomized 60 patients, with 58 ultimately receiving treatment. While the monotherapy arm was discontinued early due to futility, the combination arm met the significance criteria, demonstrating a median PFS of 3.7 months and an ORR of 19%. Exploratory analyses within the combination arm revealed differences in PFS and ORR between HPV^+^ and HPV^–^ subgroups, suggesting the potential importance of HPV status in patient selection (71). Overall, the ficlatuzumab-cetuximab combination exhibited promising outcomes, warranting further exploration in phase III trials, with HPV^–^ HNSCC potentially serving as a selection criterion (ClinicalTrials.gov identifier: NCT03422536) (71).

#### 4.1.3 TAK-701 (Galaxy Biotech)

TAK-701 is a humanized monoclonal antibody known for its high affinity to HGF. When combined with gefitinib, a small-molecule EGFR inhibitor, TAK-701 effectively suppresses the phosphorylation of both c-MET and EGFR, along with their downstream signaling pathways, in HCC827-HGF tumor cells. These cells are engineered human NSCLC cells with an activating EGFR mutation and stable expression of HGF (72). Additionally, the combined treatment of TAK-701 and gefitinib significantly inhibits tumor growth in HCC827-HGF xenograft models. These results suggest that the combined use of TAK-701 and gefitinib might offer a solution to combat resistance to EGFR-tyrosine kinase inhibitors in HGF-induced NSCLC (72). However, no significant differences between treated and control tumors were observed in event-free survival (EFS) distribution. Additionally, no objective responses were noted in any tested solid tumor xenografts (73).

#### 4.1.4 Onartuzumab (MetMAb™)

Onartuzumab, a humanized, monovalent monoclonal antibody engineered to target c-MET, operates through the knob-into-hole technology, facilitating a precise one-to-one interaction with the receptor. Its robust ability to block HGF binding, c-MET phosphorylation, and subsequent signaling within the HGF/c-MET pathway is complemented by pharmacokinetics resembling antibodies (74). Preclinical xenograft studies have shown anti-cancer solid activity for onartuzumab. Activated HGF/c-MET signaling is linked to poor prognosis and resistance to EGFR inhibitors in NSCLC (74). In a Phase II study, patients positive for c-MET who received both erlotinib and onartuzumab experienced extended PFS (2.9 months versus 1.5 months) and OS (12.6 months versus 3.8 months) in comparison to those treated solely with erlotinib (75). Nevertheless, a subsequent randomized Phase III trial could not reproduce the effectiveness shown in the Phase II trial, as there was no discernible improvement in OS (6.8 months versus 9.1 months) or PFS (2.7 months versus 2.6 months) among c-MET^+^ patients receiving combination therapy (76).

On the other hand, the phase II study GO27819 aimed to assess the efficacy of the monovalent MET inhibitor onartuzumab in combination with bevacizumab (Ona+Bev) versus placebo plus bevacizumab (Pla+Bev) for recurrent glioblastoma, considering the reported aberrant expression of c-MET in glioblastoma. Bevacizumab-naïve patients were randomized to receive either Ona+Bev or Pla+Bev until disease progression, with PFS as the primary endpoint. Secondary endpoints included OS, ORR, response duration, and safety evaluations (77).

Further investigation into biomarker subgroups is recommended despite the lack of additional clinical benefit of adding onartuzumab to bevacizumab in unselected patients with recurrent glioblastoma (77). Despite the setback in the Phase III trial, Genentech continues to explore onartuzumab’s potential in two additional Phase III trials targeting different subgroups of NSCLC, including c-MET^+^ stage IIIB or IV NSCLC with activating EGFR mutation. Subgroup analyses are expected to aid in more selective patient targeting. Furthermore, another Phase III clinical trial is underway in gastric cancer to assess the efficacy and safety of onartuzumab when administered in combination with mFOLFOX6 for treating metastatic HER2^–^ and c-MET^+^ gastroesophageal cancer (78).

Besides, the phase III trial evaluating onartuzumab plus erlotinib versus erlotinib alone as a second and third-line treatment for NSCLC did not achieve its primary endpoint of OS. This study investigated whether doses higher than the phase III dose of 15 mg/kg could enhance efficacy without compromising safety. Data from 636 patients across phase II and III NSCLC trials were analyzed. Tumor growth inhibition (TGI) models were employed to estimate individual TGI metrics, including time-to-tumor re-growth (TTG) (79). Cox regression models were developed to explore relationships between time-to-event endpoints (PFS, OS, and TTG) and baseline prognostic factors along with onartuzumab exposure. Logistic regression was used to model adverse event incidence. The analysis revealed that higher onartuzumab exposure correlated with longer PFS but not OS (79). TTG was the sole TGI metric retained in the final OS model, with onartuzumab exposure showing no significant association after adjusting for prognostic factors. However, higher onartuzumab exposure did not significantly improve OS after accounting for prognostic factors and TTG, and a trend toward increased infusion reactions and peripheral edema was noted, albeit with unknown clinical significance. These findings do not support the exploration of higher onartuzumab doses (79).

#### 4.1.5 Emibetuzumab (LY-2875358; Eli Lilly)

Emibetuzumab is a humanized, bivalent anti-c-MET antibody designed to hinder ligand-dependent and ligand-independent c-MET activation. In instances of HGF-dependent c-MET activation, emibetuzumab obstructs HGF binding to c-MET, inhibiting c-MET phosphorylation and subsequent tumor growth suppression in both *in vitro* and *in vivo* settings. This mechanism mirrors a humanized, one-armed 5D5 anti-c-MET antibody (the precursor of onartuzumab). Conversely, when c-MET activation occurs independently of HGF due to *MET* gene amplification in tumors, emibetuzumab facilitates internalization and degradation of c-MET. Treatment with emibetuzumab induces reductions in phosphorylated and total c-MET levels, leading to the inhibition of cell proliferation and tumor growth in gastric cancer cell lines (MKN-45 and SNU-5) as well as in NSCLC cell lines (EBC-1 and H1993). However, the one-armed 5D5 antibody does not exhibit anti-tumor activity in HGF-independent c-MET activation cases (80).

In a Phase I trial, emibetuzumab treatment, either alone or combined with erlotinib, resulted in sustained partial responses in NSCLC and demonstrated favorable safety and tolerability profiles. Based on pharmacokinetic/pharmacodynamic data, the recommended Phase II dose for emibetuzumab administered intravenously is 750 mg once every two weeks, either as a monotherapy or combined with erlotinib (81). Furthermore, a phase II trial sought to determine if combining emibetuzumab with erlotinib could counteract acquired resistance to erlotinib in NSCLC patients positive for protein. Patients with Stage IV NSCLC who had developed resistance to erlotinib and tested positive for c-MET were randomly assigned to receive emibetuzumab 750 mg every two weeks with or without erlotinib 150 mg once daily. The primary goal was to evaluate the overall response rate (ORR) compared to a historical control, with an additional objective focusing on ORR in patients exhibiting *MET* expression in ≥ 60% of cells. Among the 111 c-MET^+^ patients enrolled, 89 had received prior erlotinib treatment. The ORR was 3.0% for emibetuzumab plus erlotinib and 4.3% for emibetuzumab alone among patients with available post-erlotinib progression biopsies. Similar outcomes were observed in patients with ≥ 60% *MET* expression (82). Although emibetuzumab plus erlotinib showed higher disease control rates and PFS, no unexpected safety concerns were identified. Partial responses were observed regardless of EGFR mutation status or *MET* amplification. Retrospective analysis revealed EGFR-sensitizing mutations in most patients with available tissue. In summary, emibetuzumab, combined with erlotinib, did not reverse acquired resistance to erlotinib in c-MET^+^ NSCLC patients, although some patients experienced clinical benefit from the treatment (82).

#### 4.1.6 ARGX-111 (arGEN-X)

ARGX-111 is an antagonistic anti-c-MET antibody devoid of fucose, known for its robust anti-cancer efficacy due to enhanced antibody-dependent cellular cytotoxicity. A Phase Ib trial commenced to assess ARGX-111’s potential in treating advanced cancers marked by c-MET overexpression. This study focused on engineering an antagonistic anti-c-MET antibody capable of blocking HGF/c-MET signaling and inducing the killing of *MET*-overexpressing cancer cells through antibody-dependent cellular cytotoxicity (ADCC). To assess its efficacy, this ADCC-enhanced antibody with a control antibody lacking ADCC activity but capable of blocking HGF/c-MET activity was compared. Across various mouse models of cancer, including both HGF-dependent and -independent tumor xenografts, the ADCC-enhanced antibody demonstrated superior efficacy to the ADCC-inactive counterpart (83). Specifically, in orthotopic mammary carcinoma models, ADCC enhancement was crucial for depleting circulating tumor cells and suppressing metastases. Encouraged by these findings, it was further optimized the ADCC-enhanced antibody for clinical development, creating ARGX-111, which displayed improved pharmacologic properties. ARGX-111 effectively competes with HGF for c-MET binding, inhibited ligand-dependent c-MET activity, downregulated cell surface expression of c-MET, and curbed HGF-independent c-MET activity (83).

Moreover, ARGX-111 engaged NK cells to target and kill *MET*-expressing cancer cells, demonstrating c-MET-specific cytotoxic activity. ADCC assays further confirmed the cytotoxic effects of ARGX-111 across multiple human cancer cell lines and patient-derived primary tumor specimens, including cancer stem-like cells expressing c-MET. Overall, these results underscore the therapeutic advantage of ADCC over conventional HGF/c-MET signaling blockade and provide compelling evidence for the clinical evaluation of ARGX-111 in c-MET^+^ oncologic malignancies (83).

#### 4.1.7 Telisotuzumab (ABT-700)

c-MET can be activated by HGF-dependently and independently (5). Monovalent antibodies targeting c-MET/HGF interaction, such as MetMAB, have advanced clinically; however, developing therapeutic antibodies targeting c-MET has been difficult due to inherent agonistic activity independent of HGF availability (84). To address this, the bivalent anti-c-MET monoclonal antibody telisotuzumab was generated and evaluated for its binding potency, antagonistic activity, and antitumor efficacy, which can target c-MET with and without ligand. Telisotuzumab effectively blocks both HGF-independent constitutive and HGF-dependent c-MET signaling, leading to apoptosis in cancer cells dependent on amplified c-MET signaling. In preclinical models of gastric and lung cancers with c-MET amplification, telisotuzumab induced tumor regression, and delayed growth, with enhanced effects combined with chemotherapeutics. These findings, supported by fluorescence *in situ* hybridization (FISH) analysis of c-MET amplification in human cancer tissues, suggest telisotuzumab’s potential clinical benefit for treating cancers with c-MET amplification (85, 86).

The pharmacokinetics, safety, and preliminary efficacy of telisotuzumab, an antagonistic antibody targeting c-MET, were evaluated in this first-in-human phase I study. In the dose-escalation phase (3 + 3 design), 3 to 6 patients with advanced solid tumors were enrolled in four dose cohorts (5-25 mg/kg). Patients were prospectively selected for c-MET amplification using FISH screening for the dose-expansion phase. Telisotuzumab was administered intravenously on day 1 of a 21-day cycle, with 15 mg/kg selected for dose expansion based on safety and pharmacokinetic data. A total of 45 patients received at least one dose (15 in dose escalation and 30 in dose expansion). Telisotuzumab exhibited a linear pharmacokinetic profile, with peak plasma concentrations proportional to the dose level. No acute infusion reactions or dose-limiting toxicities were observed. The most common treatment-related adverse events were hypoalbuminemia (20.0%) and fatigue (11.1%). Among patients with *MET*-amplified tumors, 40.0% (4 of 10) had a confirmed partial response, 20.0% had stable disease, 30.0% had progressive disease, and one patient was not evaluable. No objective responses were observed in patients with non-amplified tumors, although 11 achieved stable disease. Telisotuzumab demonstrated an acceptable safety profile and clinical activity in patients with *MET*-amplified advanced solid tumors (87).

The ongoing development of new anti-c-MET antibodies has shown promise. The one-armed anti-c-MET antibody 2E6 successfully blocked the interaction between HGF and c-MET, thereby inhibiting subsequent signal transduction events such as the phosphorylation of c-MET, GAB1, ERK1/2, and AKT in the HepG2 HCC cell line. This antibody also significantly suppressed autocrine stimulation of HepG2 cell proliferation and HGF-induced HCC cell migration. Furthermore, it reduced HGF-induced proliferation and tube formation in human umbilical vein endothelial cells (HUVECs). In a xenograft nude mouse model, treatment with the one-armed anti-c-MET antibody significantly inhibited tumor growth in HepG2-bearing mice. These findings indicate that the one-armed anti-c-MET antibody, derived from the full-length bivalent anti-c-MET antibody, could be a potential antitumor agent for HCC (88).

**Figure 5:**
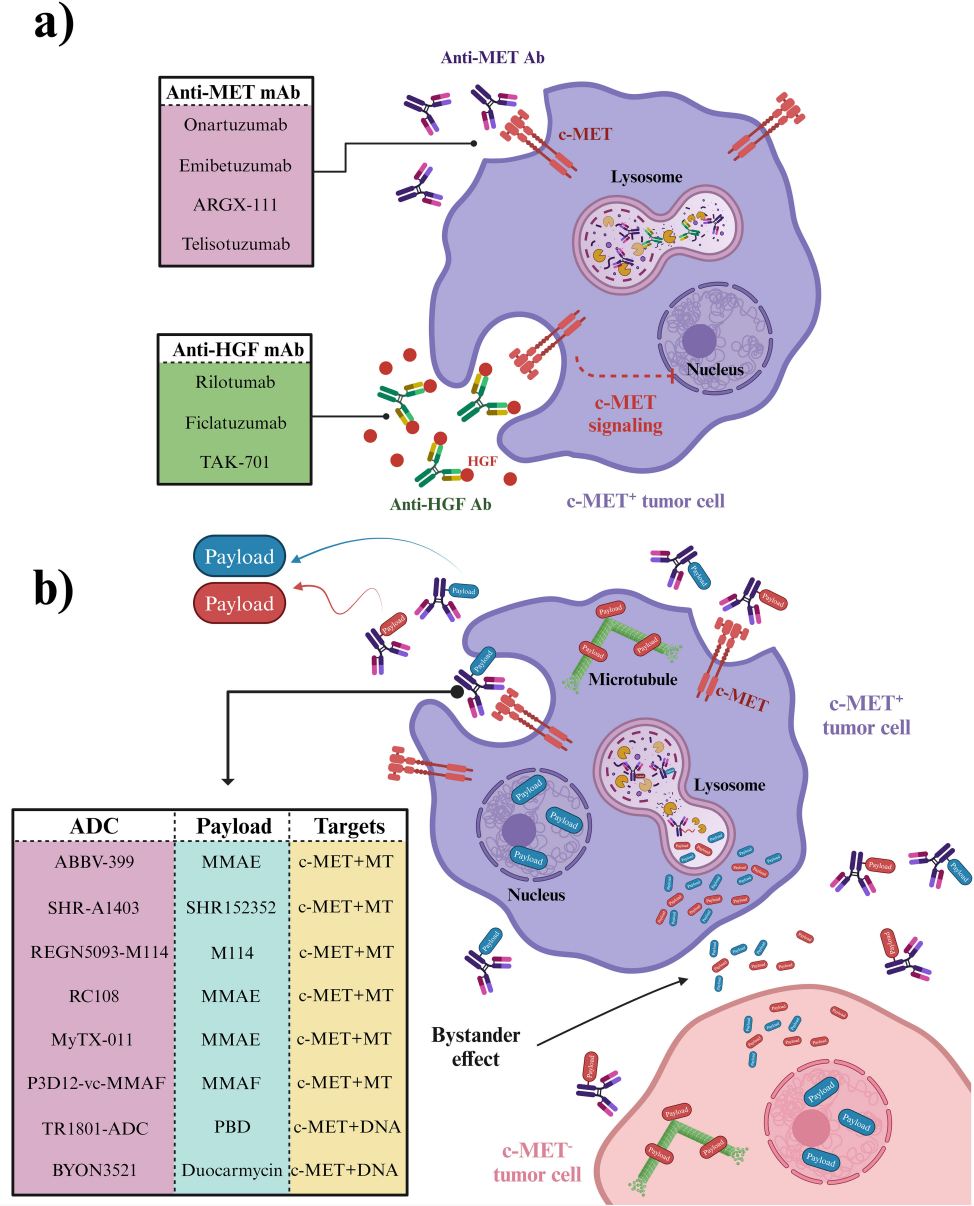
Antibodies and ADCs developed to combat c-MET signaling. **(A)** Mechanism of Monoclonal Antibodies Targeting c-MET Signaling. Anti-MET and anti-HGF antibodies specifically target c-MET signaling in c-MET^+^ cancer cells, leaving c-MET^-^ cells, including normal cells, unaffected. **(B)** Mechanism of Anti-MET ADCs. ADCs (antibody-drug conjugates) are targeted therapies that deliver chemotherapy payloads directly to c-MET^+^ cancer cells in heterogeneous cancer tissue. These payloads are then released after ADC internalization in c-MET^+^ cancer cells and distributed in the tumor microenvironment through a bystander effect, impacting c-MET^-^ cancer cells as well. *Created in BioRender. Jabbarzadeh Kaboli, P. (2024)**https://BioRender.com/p92y466*.

In summary, advancing novel anti-c-MET antibodies presents a promising avenue for cancer treatment (**Figure 5A**). Rilotumumab showcased notable enhancements in PFS and OS among patients with heightened *MET* expression in gastric and esophagogastric junction cancers. Ficlatuzumab demonstrated efficacy when paired with EGFR inhibitors across various advanced solid tumors, including NSCLC. Telisotuzumab exhibited clinical efficacy in patients with *MET*-amplified advanced solid tumors, indicating its potential therapeutic value. Furthermore, investigations into ARGX-111 and emibetuzumab emphasized the significance of targeting c-MET^+^ malignancies. The one-armed anti-c-MET antibody 2E6 also exhibited substantial antitumor effects in HCC models, suggesting its viability as a therapeutic option. These advancements underscore the diverse strategies and promising outcomes in addressing cancer through targeting the HGF/c-MET pathway.

### 4.2 Anti-MET bispecific antibodies

Bispecific antibodies (BsAb) bind to two epitopes on the same or different antigens. The secondary specificity of c-MET BsAbs, targeted at immune cell receptors such as CD3 or PD-1, is intended to use the cytotoxic capabilities of immune cells against tumor cells. CD3, situated on the surface of T cells, is pivotal in the immune response to cancer. When bound to CD3, c-MET BsAbs attract T cells toward tumor cells with heightened c-MET levels, activating and destroying tumor cells. Similarly, PD-1, found on T cells, can have its activity dampened by its interaction with its ligand PD-L1, enabling tumors to evade immune detection. By targeting PD-1 with a BsAb, the inhibition of PD-1/PD-L1 interaction can unleash T cell function, strengthening its ability to combat tumors. Consequently, the dual specificity of c-MET BsAbs toward both c-MET on tumor cells and immune cell receptors like CD3 or PD-1 facilitates the mobilization and activation of immune cells to eliminate c-MET-overexpressing tumor cells, thereby amplifying the effectiveness of cancer immunotherapy (16, 89).

#### 4.2.1 BsAb targeting c-MET and CD3

While traditional antibody-based approaches have shown limited clinical efficacy, developing immunotherapy strategies, such as c-MET/CD3 BsAbs, presents a promising alternative. The newly developed BsAb BS001 was designed to bind c-MET and CD3, demonstrating potent T cell mediated killing of tumor cells *in vitro*. Moreover, BS001 inhibited c-MET phosphorylation and downstream signaling, revealing its dual mechanism of action (89). *In vivo* experiments using lung cancer and ovarian cancer xenograft models showed effective inhibition of tumor growth by BS001, accompanied by an increase in activated tumor-infiltrating lymphocytes. Additionally, the anti-tumor effects of BS001 were enhanced by combining it with PD-L1 antibodies, suggesting a synergistic treatment approach. Nevertheless, despite the significant efficacy demonstrated by BS001, tumor recurrence was observed in some instances, underscoring the need for ongoing optimization and exploration of combination therapies to maximize therapeutic benefits (89).

#### 4.2.2 BsAbs targeting c-MET and PD-1/PD-L1

A novel BsAb was designed to target both PD-1 and c-MET, proteins implicated in cancer progression and immune response inhibition, respectively. c-MET, when deregulated, is associated with poor prognosis in many malignancies, while blocking PD-1 and PD-L1 interactions has shown promise in cancer immunotherapy. The BsAb gene, based on original PD-1 and c-MET mAb sequences, was cloned into the pCEP4 vector and expressed in 293E cells (16). The purified BsAb demonstrated simultaneous binding to PD-1 and c-MET with affinities of 11.5 nM and 9.09 nM, respectively. Functionally, the BsAb enhanced interferon (IFN)-γ production by 2-3 folds compared to control IgG, inhibited c-MET pathway activation, significantly reduced tumor cell proliferation, and exhibited dose-dependent cytotoxicity against MKN45 cells. These results suggest that the BsAb can redirect T cells to kill tumor cells while restoring T cell function and inhibiting tumor growth, indicating its potential as a therapeutic candidate for treating various solid tumors (16).

It was also discovered that BsAb can trigger the degradation of c-MET protein in cancer cells, encompassing both MKN45, a gastric cancer cell line, and A549, a lung cancer cell line. Furthermore, HGF-induced cell proliferation, migration, and antiapoptotic effects were effectively inhibited by BsAb, and HGF-stimulated phosphorylation of c-MET, Akt, and ERK1/2 was downregulated. Additionally, BsAb demonstrated the ability to restore T cell activation. Additionally, analysis using xenograft models showed that BsAb markedly inhibited the growth of tumors implanted subcutaneously and reduced chronic inflammation. These results imply the discovery of a promising bispecific therapeutic candidate that can efficiently target c-MET and PD-1 for treating human solid cancers (90). However, alongside investigating the intrinsic tumor mechanisms using molecular biology assays *in vitro*, a humanized mouse model was utilized to assess the BsAb’s anti-tumor activity *in vivo*. The ability of this BsAb to inhibit the migration of c-MET/PD-L1^+^ CRC cells and exhibit robust anti-tumor effects against HCT116 tumors in mice was demonstrated, possibly by inducing the degradation of c-MET protein in a dose and time-dependent manner (39).

Additionally, the suppression of phosphorylation of downstream c-MET proteins GAB1 and focal adhesion kinase (FAK) was observed with the BsAb. Concerning the extrinsic tumor mechanism, macrophage-mediated phagocytosis may be enhanced by the BsAb. The BsAb demonstrated potent anti-tumor effects through two distinct mechanisms: inhibition of c-MET signal transduction and promoting macrophage-mediated phagocytosis; promised as a novel therapeutic option for patients with c-MET/PD-L1^+^ CRC, the status of exosomal-c-MET/PD-L1 may serve as a biomarker for predicting responsiveness to BsAb treatment (39).

#### 4.2.3 BsAb targeting c-MET and CD137

In recent cancer immunotherapy research, targeting the costimulatory receptor CD137 has emerged promising, showing anti-tumor efficacy in clinical trials. However, the initial CD137 agonistic antibodies, sarilumab and utomilumab, faced challenges due to liver toxicity and insufficient efficacy (91, 92). Additionally, c-MET has been identified as a significantly expressed tumor-associated antigen across various tumor types. Accordingly, developing a BsAb targeting both c-MET and CD137 aimed to optimize the BsAb format and CD137 binder to ensure efficient delivery of the CD137 agonist to the tumor microenvironment. A monovalent c-MET motif and a trimeric CD137 Variable Heavy domain of Heavy chain (VHH) showed promising results in BsAb design. The c-MET x CD137 BsAb was found to provide co-stimulation to T cells through cross-linking by c-MET-expressing tumor cells. Enhanced efficacy and potency, including activation of CD137 signaling, target cell killing, and cytokine release across various tumor cell lines, were confirmed through functional immune assays (93). Furthermore, a dose-dependent enhancement of target-induced T cell cytokine release was demonstrated when combining c-METxCD137 BsAb with pembrolizumab. Therefore, the c-MET x CD137 BsAb minimizes off-target effects while effectively delivering immune agonism, thus offering a solution to resistance observed in anti-PD-1/PD-L1 therapy (93).

#### 4.2.4 BsAb targeting c-MET and CTLA-4

While PD-1 primarily regulates immune responses in peripheral tissues to prevent tissue damage, cytotoxic T-lymphocyte associated protein 4 (CTLA-4) is critical in controlling T cell activation and proliferation in secondary lymphoid organs, modulating the immune response, and enhancing antitumor immunity. CTLA-4 blockade has shown efficacy in promoting the proliferation and function of effector T cells while inhibiting the suppressive activity of regulatory T cells (Tregs), contributing to immune tolerance and tumor evasion. In this context, introducing BsAb-5, a novel bispecific antibody targeting c-MET and CTLA-4 in CD166^+^ LCSCs, represents a significant advancement (94). BsAb-5 demonstrated efficacy in inhibiting HGF-mediated tumor development and inducing c-MET degradation, supported by *in vitro* assays and *in vivo* xenograft studies. Moreover, BsAb-5’s antitumor effects were associated with suppressing Tregs and the upregulation of effector T cells, suggesting its potential as a therapeutic option for human NSCLC and other malignancies (94).

#### 4.2.5 BsAb targeting c-MET and EGFR

The effectiveness of targeting multiple drug receptors with BsAbs is determined by the relative levels of these receptors on the cell surface. NSCLC develops resistance to EGFR tyrosine kinase inhibitors with mutations in EGFR through diverse mechanisms, including activating the c-MET receptor pathway. The correlation between receptor density values and the *in vitro* activity of a BsAb called JNJ-61186372, which targets both the EGFR and the c-MET, was also investigated (95). The simultaneous binding of the BsAb to both receptors was determined on a panel of 11 tumor cell lines using Quantitative Fluorescence Cytometry (QFCM). The Antibody Binding Capacity values indicate the number of antibody binding sites per cell (95). It was found that the levels of EGFR and c-MET receptor density were correlated with their respective gene expression levels and receptor phosphorylation inhibition values. Interestingly, a preference for binding to the more highly expressed receptor, whether EGFR or c-MET, was observed in the BsAb, resulting in enhanced potency against the less highly expressed target. These findings led to the proposal of an avidity model to explain how JNJ-61186372 engages both EGFR and c-MET, which may have broad implications for the efficacy and design of bispecific drugs (95).

JNJ-61186372 exhibited anti-tumor effects in wild-type and mutant EGFR and c-MET pathway activation scenarios. Engineered to have low fucosylation (<10%), it demonstrated improved antibody-dependent cell-mediated cytotoxicity and binding to FcγRIIIa (96). *In vitro* and *in vivo* studies using single-arm versions of JNJ-61186372 targeting EGFR or c-MET showed the importance of its Fc activity—binding of the anti-EGFR arm and c-MET arm in inhibiting EGFR or c-MET-driven tumor cells is facilitated by Fc suggesting that the Fc function of JNJ-61186372 is essential for maximal tumor inhibition (96). Furthermore, in the same model, downregulation of both EGFR and c-MET receptors was induced by treatment with Fc-competent JNJ-61186372, indicating the essential role of Fc interactions in receptor modulation *in vivo* and therapeutic efficacy. These Fc-mediated activities, in conjunction with the inhibition of both EGFR and c-MET signaling pathways, demonstrate the multifaceted strategy of JNJ-61186372 in addressing therapeutic resistance in EGFR mutant patients (96). In a mouse xenograft model, JNJ-61186372 demonstrated greater efficacy than the combination therapy involving monovalent antibodies targeting anti-EGFR and anti-c-MET, administered at the same dose level (97).

In summary, BsAbs targeting multiple receptors, such as c-MET, EGFR and immune cell receptors, offer a promising approach to cancer immunotherapy by leveraging immune cell cytotoxicity and restoring immune response function. These BsAbs hold the potential to overcome therapeutic resistance and improve clinical outcomes in cancer patients.

### 4.3 Anti-MET antibody-drug conjugates

Antibody-drug conjugates (ADCs) are considered a unique category of drugs consisting of a monoclonal antibody, payload, and linker, recognized for their high specificity and affinity for cell surface proteins. Upon binding to a membrane antigen, the payload, typically a toxic agent, is facilitated for internalization into cancer cell cytoplasm in response to the lysosome’s low pH by ADCs. The payload is a small molecule chemotherapy drug; it can easily cross the membrane through simple diffusion and be released into the neighboring tumor cells upon dissociation from the ADC under lysosomal conditions. The released payload then indices cytotoxic effects within the other cancer cells by targeting various cellular processes such as DNA structure, microtubule formation, or protein synthesis, potentially affecting neighboring cells through the bystander effect (98). While tumor heterogeneity may limit ADC efficacy due to lacking target antigens in some neighboring cancer cells, certain ADCs can still distribute their payloads to antigen-negative cells. However, ADCs like ado-trastuzumab emtansine (T-DM1), utilized for Her2^+^ breast cancer, are characterized by non-cleavable linkers and lack bystander effects (99). Nevertheless, ADCs remain esteemed as therapeutic vehicles for the specific delivery of highly toxic chemotherapy to the tumor microenvironment, enabling the targeting of heterogeneous cancer cells while minimizing off-target drug delivery (98). Among more than 40 clinical trials started or completed on anti-c-MET drugs, seven ADCs targeting c-MET are currently under investigation across various stages of clinical trials (**Figure 5B**).

#### 4.3.1 ABBV-399 (telisotuzumab vedotin, Teliso-V)

The ABBV-399 or telisotuzumab vedotin was developed by conjugating valine-citrulline-monomethylauristatin E (vc-MMAE) to the interchain disulfide bonds of ABT-700 after a mild reduction to the sulfhydryl groups (100). It has been demonstrated to have antitumor activity in cancer cells overexpressing c-MET or with amplified *MET*, as well as in xenografts, including patient-derived xenograft (PDX) models and those resistant to other c-MET inhibitors. It was found that a threshold level of c-MET expression in sensitive tumor cells, but not in normal cells, is necessary for significant ABBV-399-mediated tumor cell killing. c-MET or amplified *MET* cell lines and PDX models show substantial tumor growth inhibition and regressions. The growth of xenograft tumors resistant to other c-MET inhibitors is effectively inhibited by ABBV-399, which also offers significant therapeutic benefits when combined with standard chemotherapy (101). Consequently, ABBV-399 is a novel strategy to deliver a potent cytotoxin to c-MET-overexpressing tumor cells, facilitating cell killing independent of c-MET signaling. A phase I clinical study has been advanced to ABBV-399, where it has been well tolerated, and objective responses have been produced in c-MET-expressing NSCLC patients (101).

Furthermore, ABBV-399 monotherapy was evaluated in Phase I clinical trial, being administered intravenously every two weeks (1.6-2.2 mg/kg) or every three weeks (0.15-3.3 mg/kg), with a dose-expansion phase focusing on patients with c-MET^+^ NSCLC or *MET* amplification/exon 14 skipping mutations (ClinicalTrials.gov identifier: NCT02099058). Fifty-two patients received doses of ≥1.6 mg/kg biweekly or ≥2.4 mg/kg biweekly (102, 103). Fatigue, peripheral neuropathy, and nausea were the most common adverse events, with no dose-limiting toxicities being observed up to 2.2 mg/kg biweekly and 2.7 mg/kg biweekly. The recommended Phase II doses were set at 1.9 mg/kg biweekly and 2.7 mg/kg biweekly (ClinicalTrials.gov identifier: NCT03539536). Among the 40 efficacy-evaluable c-MET^+^ patients, 23% had objective responses with a median response duration of 8.7 months and a PFS of 5.2 months. ABBV-399 monotherapy was well tolerated and showed antitumor activity, supporting further clinical development at the specified dosing schedules (102).

Furthermore, the Phase III clinical trial was recently started (2022-03-25) on telisotuzumab vedotin for treating NSCLC. Participants in the study will be randomly assigned to receive either Teliso-V or Docetaxel in a 1:1 ratio. Each group will receive intravenous (IV) infusions of either telisotuzumab vedotin or docetaxel. The study aims to enroll approximately 698 adult participants with c-Met overexpressing NSCLC across roughly 300 sites worldwide (ClinicalTrials.gov identifier: NCT04928846).

In summary, ABBV-399 (telisotuzumab vedotin, Teliso-V) represents a promising advancement in targeting c-MET-overexpressing tumor cells, demonstrating significant antitumor activity across various preclinical models, including those resistant to other c-MET inhibitors. The Phase I clinical trials have yielded encouraging results, with manageable adverse effects and notable antitumor efficacy, which has propelled the compound into Phase III trials. This ongoing phase III study seeks to further evaluate the therapeutic potential of telisotuzumab vedotin in comparison to docetaxel in NSCLC patients with c-MET overexpression, aiming to solidify its role in the treatment landscape of this challenging malignancy. The trial’s broad international participation and robust design underscore the high expectations for ABBV-399 as a potentially transformative therapy in NSCLC.

#### 4.3.2 SHR-A1403 (*HTI-1066)*

SHR-A1403, a Phase 1 clinical-stage c-MET-ADC, is a novel formulation comprising a humanized anti-c-MET antibody (of IgG2 subtype) connected via a non-cleavable linker to an enhanced version of a cytotoxic microtubule inhibitor SHR152852. The aim is to address possible limitations seen with ABBV-399. *In vitro* and *in vivo* effects of SHR-A1403 were investigated, and a solid binding affinity was demonstrated to human and monkey c-MET proteins.

ABBV-399 faces several limitations despite its rapid progress. It combines the c-MET-targeting antibody ABT-700 with the toxin MMAE using a cleavable linker, resulting in a non-fixed Drug-to-Antibody Ratio (DAR) of 3.1, affecting stability and efficacy. Additionally, the safety window of MMAE is relatively narrow, increasing the risk of toxicity. In contrast, SHR-A1403 offers significant benefits, including a higher safety window—64 times greater than MMAE. It also features a more consistent DAR value close to 2 and employs non-cleavable linkers, preventing premature toxin release and reducing off-target effects. Furthermore, SHR152852’s low cell permeability enhances its safety profile, making SHR-A1403 a promising candidate for clinical trials with controllable risks (104).

Effective inhibition of cancer cell lines with high c-MET expression was observed. In mouse models with tumors from cell lines or patient tissues exhibiting c-MET overexpression, robust anti-tumor activity was shown by SHR-A1403. The internalization of SHR-A1403 was facilitated by binding the antibody to c-MET, leading to subsequent lysosomal translocation and cytotoxicity of the released toxin, likely serving as the primary mechanisms underlying its anti-tumor effects (105). Overall, significant anti-tumor activity in various preclinical models with high c-MET levels was demonstrated by SHR-A1403, suggesting its potential as a therapeutic option for c-MET-overexpressing cancers. Further, a novel strategy was proposed to combat resistance to the EGFR-tyrosine kinase inhibitor (TKI), AZD9291, in NSCLC cells using SHR-A1403 (106). However, crizotinib and the c-Met monoclonal antibody AZD9291 alone could not overcome resistance to c-MET targeted therapy in cells overexpressing c-MET. On the other hand, the combination of AZD9291 and crizotinib partially reversed resistance in cells with elevated phospho-c-MET levels by synergistically inhibiting downstream targets.

In addition, potent inhibition of proliferation in AZD9291-resistant cells overexpressing c-MET was exhibited by SHR-A1403, regardless of c-MET phosphorylation levels. SHR-A1403 was internalized into resistant cells and released the associated microtubule inhibitor, leading to cell-killing activity dependent solely on c-MET expression levels, irrespective of c-MET or EGFR signaling involvement in AZD9291 resistance. Consistent with its *in vitro* efficacy, significant suppression of the growth of AZD9291-resistant HCC827 tumors and induction of tumor regression *in vivo* were observed with SHR-A1403. These findings highlight the potential of SHR-A1403 to overcome AZD9291 resistance in cells with elevated c-MET expression, suggesting c-MET expression as a predictive biomarker for SHR-A1403 efficacy (106).

Additionally, SHR-A1403 has shown potential as a targeted treatment for pancreatic ductal adenocarcinoma (PDAC) with high c-MET expression. The first study examining SHR-A1403 in preclinical PDAC models revealed that it inhibited pancreatic cancer cell proliferation, migration, and invasion while also inducing cell cycle arrest and apoptosis (104). These effects were linked to SHR-A1403’s inhibition of intracellular cholesterol biosynthesis. As a result, SHR-A1403 demonstrated strong preclinical anti-tumor efficacy in pancreatic cancer, indicating its potential use as a c-MET-targeted antibody-drug conjugate treatment for PDAC in clinical practice (104).

Besides, the PK of SHR-A1403 was meticulously examined *in vivo* in mice, rats, and monkeys (107). ELISA methods were employed to measure serum levels of both the ADC and total antibody, revealing a prolonged half-life (t1/2) of ADC compared with total antibody in the serum ranging from 4.6 to 11.3 days across species, indicating low systemic clearance. Studies in tumor-bearing mice demonstrated that ^125^I-SHR-A1403 accumulated notably in tumor tissues compared to other organs, highlighting its favorable safety profile and attributes as an ADC. Monkeys displayed minimal changes in PK profiles despite a relatively low anti-drug antibody (ADA) level. Variations in exposure and ADA incidence during the discovery phase, depending on different DAR for SHR-A1403, led to selecting an optimal DAR value (DAR = 2) for further development. Overall, the PK characterization of SHR-A1403 yielded favorable results, supporting its investigational new drug application and ongoing first-in-human trial in the US (ClinicalTrials.gov identifier: NCT0385654) (107).

#### 4.3.3 TR1801-ADC

A novel ‘third generation’ c-MET-targeted ADC, TR1801-ADC, was developed with enhancements in specificity, stability, toxin-linker, conjugation site, and *in vivo* efficacy (108). Picomolar activity against cancer cell lines from various solid tumors, including lung, colorectal, and gastric cancers, was exhibited by this nonagonistic c-MET antibody linked to the pyrrolobenzodiazepine (PBD) toxin-linker tesirine. High antitumor activity was demonstrated by TR1801-ADC in both high and medium-to-low c-MET-expressing cell lines, independent of *MET* gene copy number, outperforming a c-MET-ADC with a tubulin inhibitor payload. Significant responsiveness to TR1801-ADC was revealed *in vivo* studies of several xenograft models generated using cancer cell lines with low-to-medium c-MET expression, even at a single dose. Furthermore, in the case of various patient-derived xenograft models, remarkable efficacy was shown by TR1801-ADC, achieving complete tumor regression in 90% of tested patient-derived xenograft models of gastric, colorectal, and head and neck cancers. Overall, superior preclinical efficacy and good tolerability in rats were demonstrated by this new generation of c-MET-ADCs (ClinicalTrials.gov identifier: NCT03859752) (108).

On the other hand, pancreatic cancer, with its aggressiveness and poor 5-year OS rate, is characterized by posing significant challenges in treatment due to late detection and limited response to chemotherapy. TR1801-ADC was investigated as a potential solution (109). Initial examinations revealed heightened c-MET expression at the plasma membrane of pancreatic cancer cells, prompting *in vitro* assessment of TR1801-ADC in these cell lines. Impressively, It has been demonstrated that c-MET is highly expressed and located at the plasma membrane of pancreatic cancer cells. Specific cytotoxicity was induced in pancreatic cancer cell lines by TR1801-ADC, resulting in profound tumor growth inhibition, even in gemcitabine-resistant tumors. Additionally, synergism between TR1801-ADC and gemcitabine was observed *in vitro*, and an improved response to the combination was noted *in vivo* (109).

#### 4.3.4 BYON3521

BYON3521 is an innovative duocarmycin-based ADC comprising a humanized cysteine-engineered IgG1 monoclonal antibody with a solid binding affinity for human and cynomolgus c-MET receptors. *In vitro* experiments illustrated the effective internalization of BYON3521 upon c-MET binding, leading to targeted and bystander-mediated cell death (110). The ADC demonstrated robust potency and complete efficacy in cancer cell lines with *MET* amplification and high c-MET expression. It also exhibited good potency with partial efficacy in cell lines expressing moderate to low levels of c-MET. In mouse xenograft models, a single dose of BYON3521 produced significant antitumor effects across various tumor types without *MET* amplification, achieving complete tumor regression in models with moderate c-MET expression. In repeated-dose Good Laboratory Practice (GLP) safety assessments conducted in cynomolgus monkeys, BYON3521 was well tolerated, with the highest non-severely toxic dose established at 15 mg/kg, based on safety data from cynomolgus monkeys, a human PK model estimated the minimal efficacious dose in humans to be between 3 to 4 mg/kg (110). Overall, preclinical data suggests that BYON3521 is a safe ADC with promising clinical potential. A phase I dose-escalation study is ongoing to determine the maximum tolerated dose and recommended dose for expansion (ClinicalTrials.gov identifier: NCT05323045).

#### 4.3.5 REGN5093-M114

To address the issue of resistance to EGFR-TKI in EGFR-mutated NSCLC patients, the preclinical effectiveness of REGN5093-M114, a novel antibody-drug conjugate targeting c-MET in c-MET-driven patient-derived models, was recently investigated (111). A biparatopic METxMET antibody (REGN5093), wherein each arm of the antibody recognizes a distinct epitope of c-MET, was initially created. Subsequently, REGN5093-M114 was developed by conjugating a novel maytansinoid M114 payload to REGN5093 (112). *In vitro* and *in vivo* evaluations of REGN5093-M114 were conducted using patient-derived organoids, patient-derived cells, or ATCC cell lines. It was found that REGN5093-M114 displayed significant antitumor efficacy compared to c-MET-TKI or unconjugated METxMET biparatopic antibody (REGN5093). Notably, treatment with REGN5093-M114 showed favorable responses in TKI-naïve EGFR-mutant NSCLC cells with c-MET overexpression, irrespective of MET gene copy number (111). Additionally, the expression of c-MET on the cell surface emerged as a predictive indicator for the efficacy of REGN5093-M114. Furthermore, REGN5093-M114 effectively reduced tumor growth in EGFR-mutant NSCLC cases with PTEN loss or c-MET-Y1230C mutation, even in cases of prior progression on osimertinib and savolitinib treatment (111). These findings suggest that REGN5093-M114 holds potential as a candidate for addressing the challenges associated with functional c-MET pathway blockade., which led to the launching of the first Phase I/II clinical trial on REGN5093-M114 ADC (ClinicalTrials.gov identifier: NCT04982224).

#### 4.3.6 RC108

RC108, an ADC developed by RemeGen, consists of an anti-c-MET monoclonal antibody linked to the antimicrotubule agent MMAE via a cleavable vc-Linker (113). In November 2020, RemeGen was approved by the National Medical Products Administration to initiate Phase 1 clinical trials of RC108 in c-MET^+^ advanced solid tumors in China (ClinicalTrials.gov identifiers: NCT04617314). In December 2022, RC108 received clinical trial authorization from the U.S. FDA to research c-MET^+^ solid tumors (ClinicalTrials.gov identifiers: NCT05628857). By April 2023, RC108 had been approved for Phase 1b/2 clinical research in China targeting locally advanced or metastatic NSCLC with EGFR mutations that had failed c-MET expression treatment with EGFR-TKIs (ClinicalTrials.gov identifiers: NCT05821933). The results of the preclinical and clinical studies for RC-108 ADC were not publicly disclosed.

#### 4.3.7 MYTX-011

Advances in linker payload technology and target selection have significantly enhanced ADC design, leading to several approvals over the past decade. The potential of incorporating pH-dependent binding in the antibody component of MYTX-011, a c-MET-targeting ADC, to bypass the need for high c-MET expression on tumors was demonstrated, potentially benefiting a broader patient population with lower c-MET levels (114). MYTX-011 achieved four-fold higher net internalization than a non-pH-engineered parent ADC in NSCLC cells, and increased cytotoxicity was demonstrated against various solid tumor cell lines. In mouse xenograft models of NSCLC with varying c-MET expression levels, at least three-fold higher efficacy was exhibited by a single dose of MYTX-011 compared to a benchmark ADC. Additionally, improved pharmacokinetics were shown by MYTX-011 compared to parent and benchmark ADCs. In a repeat dose toxicology study, a toxicity profile similar to other MMAE-based ADCs was exhibited by MYTX-011 (114). These findings suggest that MYTX-011 can treat a broader range of NSCLC patients with c-MET expression compared to other c-MET-targeting ADCs. A first-in-human study is currently underway to evaluate the safety, tolerability, and preliminary efficacy of MYTX-011 in NSCLC patients (ClinicalTrials.gov identifiers: NCT05652868).

#### 4.3.8 P3D12-vc-MMAF

Responses to c-MET inhibitors have been seen in clinical trials; however, their effectiveness seems restricted to cases involving MET gene amplifications or mutations. A c-MET targeted ADC was developed, exhibiting preclinical activity without MET gene amplification or mutation and showing efficacy in moderate protein expression. This ADC utilizes a high-affinity c-MET antibody (P3D12) that induces c-MET degradation with minimal activation of c-MET signaling or mitogenic effects (115). The P3D12 antibody was conjugated to vc-Monomethylauristatin F (MMAF). Potent *in vitro* activity was demonstrated by P3D12-vc-MMAF in c-MET protein-expressing cell lines regardless of MET gene status, maintaining activity in cell lines with medium-low c-MET protein expression. These data suggest that P3D12-vc-MMAF may offer a superior clinical profile for treating c-MET^+^ malignancies compared to c-MET pathway inhibitors (115).

The development and evaluation of c-MET-targeting ADCs have demonstrated their potential as effective therapeutic agents for treating various c-MET^+^ malignancies. These ADCs leverage the specificity of monoclonal antibodies to deliver potent cytotoxins directly to cancer cells, minimizing off-target effects and enhancing antitumor activity. The preclinical and early clinical successes of ADCs like ABBV-399, SHR-A1403, and others underscore their ability to address unmet needs in oncology, particularly in tumors with moderate c-MET expression and resistance to other treatments. Combining ADCs with other therapies further amplifies their therapeutic potential.

Overall, the promising results from these studies highlight the significant clinical potential of c-MET-targeting ADCs, paving the way for their continued development and potential approval as targeted cancer therapies. Ongoing and future clinical trials will be critical in confirming their efficacy and safety profiles, benefiting a broader range of patients with c-MET^+^ tumors.

## 5 Anti-MET chimeric antigen receptors T/NK cell therapy

**Figure 6:**
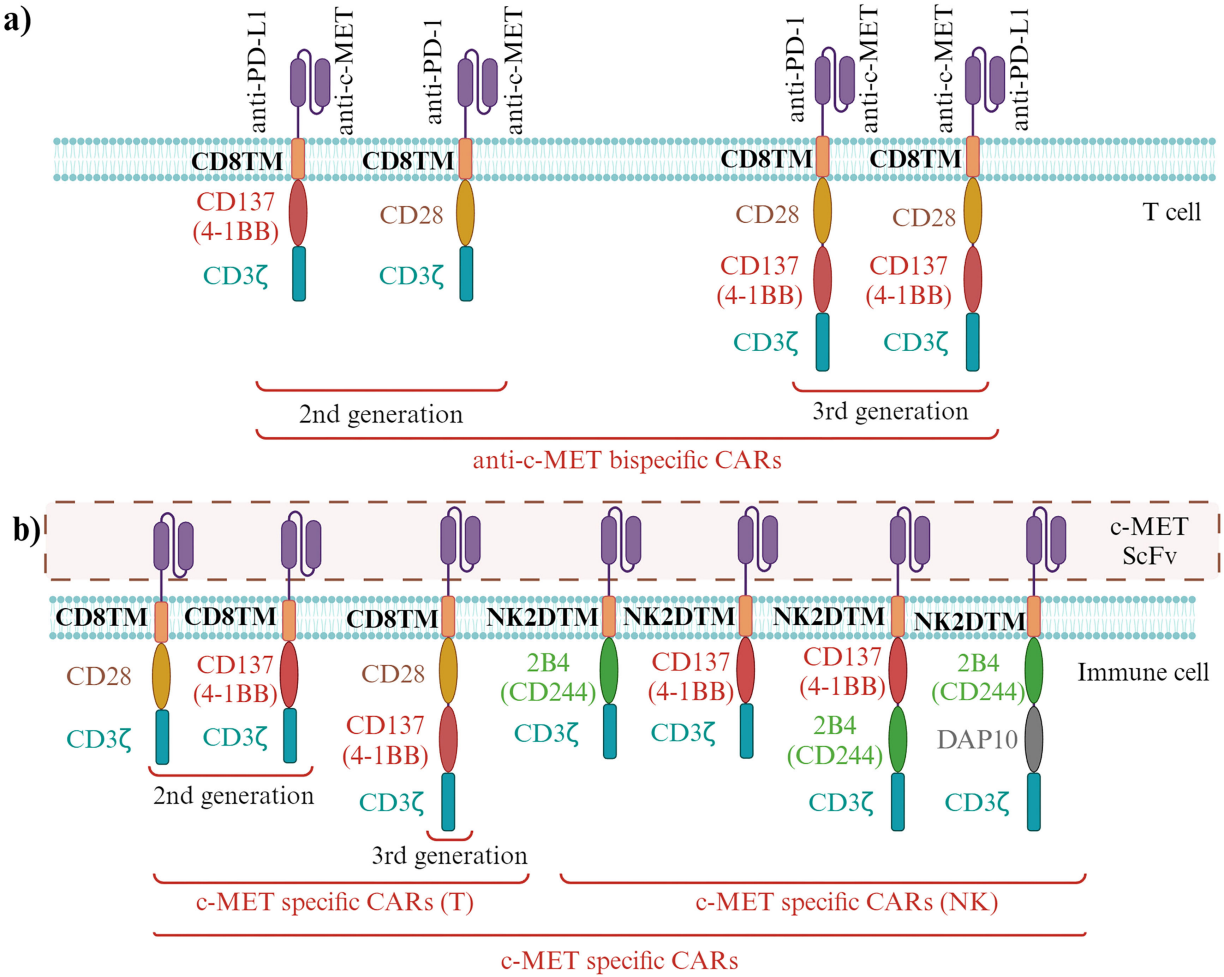
Specialized immunotherapeutic approaches targeting c-MET (10, 17, 116–120). **(A)** Immunotherapeutic Strategies Antagonizing c-MET. Development of bispecific c-MET antagonizing CARs that simultaneously target cells bearing PD-1 and PD-L1. **(B)** c-MET-Specific CARs Designed to Recognize c-MET^+^ Cells, Triggering T Cells and NK Cells Against c-MET. Various c-MET-specific CARs have been engineered, with distinct transmembrane domains utilized depending on the engineered immune cells. *Created in BioRender. Jabbarzadeh Kaboli, P. (2024)**https://BioRender.com/n53g868*.

In immunotherapy, CAR-immune cells have emerged as a groundbreaking approach, bestowing the remarkable ability to confer specificity onto T, NK, and macrophage immune cells. This cellular engineering marvel equips these immune warriors to target antigen-bearing tumor cells selectively. At the heart of CARs lies a compact yet potent design featuring a single-chain variable fragment (scFv) derived from an antibody, intracellular costimulatory domains (whose composition varies with CAR generation), and transmembrane domains tailored to the immune cell type (**Figure 6A**). Consequently, CARs exhibit the capacity to recognize distinct tumor-associated antigens, culminating in five evolving generations. The initial generation, CARs with CD3ζ, evolved into the second generation, which introduced additional costimulatory domains like CD28 or 4-1BB. The third generation expanded the repertoire by incorporating yet another costimulatory domain, often a combination of CD28 and 4-1BB. Subsequent progress has yielded the fourth and fifth generations of CARs, enriched with intracellular domains linked to cytokines like interleukin (IL)-12 and IL-2Rβ, with the power to stimulate cytokine secretion.

Meanwhile, CAR-based immunotherapy has illuminated NK cells and macrophages as compelling candidates. CAR-NK cells have gained traction due to their distinct advantages, notably their independence from specific antigens, ability to evade graft-versus-host disease (GVHD), and a unique cytokine profile expression that diminishes the risk of neurotoxicity and cytokine release syndrome (CRS) (121). While peripheral blood serves as the primary source for preclinical studies, hematopoietic stem cells and umbilical cord blood have also been harnessed (122). The foundational CAR structure for NK cells mirrors that of CAR-T cells, apart from the intracellular domain, housing activating receptors such as 2B4, 4-1BB, DAP10, and DAP12 (123) (**Figure 6B**).

In solid tumor therapy, several CAR-based immunotherapeutic endeavors have set their sights on RTKs, albeit with one conspicuous omission—c-MET (**Table 2**). However, recent strides have seen the development of CAR-T and CAR-NK cell therapies with c-MET squarely in their crosshairs, paving the way for prospective clinical investigations. The emerging group of anti-c-MET CAR-T cells has exhibited remarkable efficacy against cancer cells with c-MET overexpression. Manufacturing T cells with anti-c-MET-CAR and anti-PD-1-CAR have demonstrated potent *in vitro* and *in vivo* antitumor effects. This effect is accentuated by a substantial increase in the production of crucial immune-signaling molecules such as IL-2, TNF-α, and IFN-γ when these anti-c-MET/anti-PD-1 CAR-T cells are co-cultured (120). These innovative constructs, typified by the anti-c-MET-CARs, comprise an scFv derived from an anti-c-MET antibody, CD28, and CD3ζ (responsible for signaling), with a persistent aim to inhibit c-MET in lung cancer cells.

**Table 2:** Clinical trials on CAR-immune cells in RTK^+^ solid tumors.

ID	Title	Targeted RTK	Dates	Phase
NCT05341492	EGFR/B7H3 CAR-T cell on Lung Cancer and Triple Negative Breast Cancer	EGFR	2022-05-01 ^S^	Early 1
NCT02873390 ^U^ NCT02862028 ^U^	PD-1 Antibody Expressing CAR-T Cells for EGFR Family Member Positive Advanced Solid Tumor	EGFR	2016-08 ^S^ 2018-07 ^C^	1/2
NCT03182816 ^U^	CTLA-4 and PD-1 Antibodies Expressing EGFR-CAR-T Cells for EGFR^+^ Advanced Solid Tumor	EGFR	2017-06-07 ^S^ 2019-04-20 ^C^	1/2
NCT03618381	EGFR806 CAR-T Cell Immunotherapy for Recurrent/​Refractory Solid Tumors in Children and Young Adults	EGFR	2019-06-18 ^S^	1
NCT04976218	TGFβR-KO CAR-EGFR T Cells in Previously Treated Advanced EGFR^+^ Solid Tumors	EGFR	2022-03-15 ^S^	1
NCT03740256	Binary Oncolytic Adenovirus in Combination with HER2-Specific Autologous CAR VST, Advanced HER2^+^ Solid Tumors (VISTA)	HER2	2020-12-14 ^S^	1
NCT04650451	Safety and Activity Study of HER2-Targeted Dual Switch CAR-T Cells (BPX-603) in Subjects with HER2^+^ Solid Tumors	HER2	2020-12-07 ^S^	1
NCT05681650	HER2 Targeted hypoxia-stimulated CAR-T Cells in HER2^+^ Advanced Solid Tumors	HER2	2023-02-01 ^S^	1/2
NCT05631899	Combination of CAR-DC cell Vaccine and Anti-PD-1 Antibody in Local Advanced/​Metastatic Solid Tumors	EphA2	2023-02-03 ^S^	1
NCT05631886	Combination of CAR-DC cell Vaccine and Anti-PD-1 Antibody in Malignant Tumors	EphA2	2023-02-06 ^S^	1
NCT05477927	Dual-targeting VEGFR1 and PD-L1 CAR-T cells for cancer patients with Pleural or Peritoneal Metastases	VEGFR1	2022-10-30 ^S^	1
NCT02706392 ^T^	Genetically Modified T Cell Therapy in Treating Patients with Advanced ROR1^+^ Malignancies	ROR1	2016-03-16 ^S^ 2021-09-28 ^C^	1

U, Unknown (status); T, Terminated (status); S, Started (date); C, Completed (date).

Furthermore, the application of anti-c-MET-CAR-T cells and CAR-NK cells against gastric cancer cells and glioblastoma has unveiled their potential, exemplified by enhanced IL-2 secretion and cytotoxicity against c-MET^+^ cancer cells in a xenograft mouse model hosting MKN-45 metastatic gastric cancer cells with c-MET amplification, c-MET-CAR-T cells demonstrated substantial impact (124) (**Figure 6B**).

A compelling contrast emerges between monovalent CAR-T cells targeting either anti-c-MET or anti-PD-L1 and their bivalent counterparts, the c-MET/PD-L1-CAR-T cells. The latter exhibit significantly elevated antitumor activity against HCC *in vivo*, marked by heightened secretion of IFN-γ and IL-2 by T cells in response to c-MET^+^ and PD-L1^+^ tumor cells (125). Further investigation into the efficacy of 2nd and 3rd-generation c-MET-CARs has reaffirmed their potential. The study reveals that both generations of c-MET-specific CAR-T cells, bearing intracellular domains of 4-1BB or CD28, stably expressed on T cell membranes, effectively target c-MET^+^ HCC cells *in vitro* and *in vivo*. However, the 3rd generation, featuring a combination of 4-1BB and CD28 intracellular domains, emerges as the frontrunner, producing higher levels of IFN-γ and IL-2 and exhibiting greater cytokine secretion when co-cultured with MET^high^ HCC cells (117).

The c-MET-targeted CAR immunotherapy extends its reach to primary NK cells, where a c-MET-specific CAR construct, incorporating the CD8α-4-1BB–DAP12 sequence, has taken center stage. This construct capitalizes on CD8α expression in CD8^+^ T cells, 4-1BB’s presence in both T and NK cells, and DAP12’s association with NK cells. The study evaluates the efficacy of c-MET-CAR-NK cells against MET^high^ HepG2 HCC and MET^low^ H1299 lung cancer cells, with striking results observed, particularly in the context of HepG2 cells (126). Another facet of this research delves into the diversification of c-MET-CAR-NK cells through the construction of four distinct c-MET-CARs, each featuring different combinations of NK-specific signaling domains, such as NKG2 transmembrane domains and intracellular domains of 2B4, 4-1BB, and DAP10. These tailored c-MET-CARs, CC1-4 and a control CD19-CAR, are then transfected into CD56^+^ CD16^-^ NK-92 cells. Their effectiveness is tested against various lung cancer cell lines *in vitro* and H1299 xenograft tumors *in vivo*. Impressively, CCN4 NK cells, fortified with DAP10, emerge as the most potent, exerting significant cytotoxicity on tumor cells *in vitro* and *in vivo*. This breakthrough study underscores the efficacy of c-MET-specific CARs in fortifying NK cells against c-MET^+^ lung adenocarcinoma (10) (**Figure 6B**).

**Figure 7:**
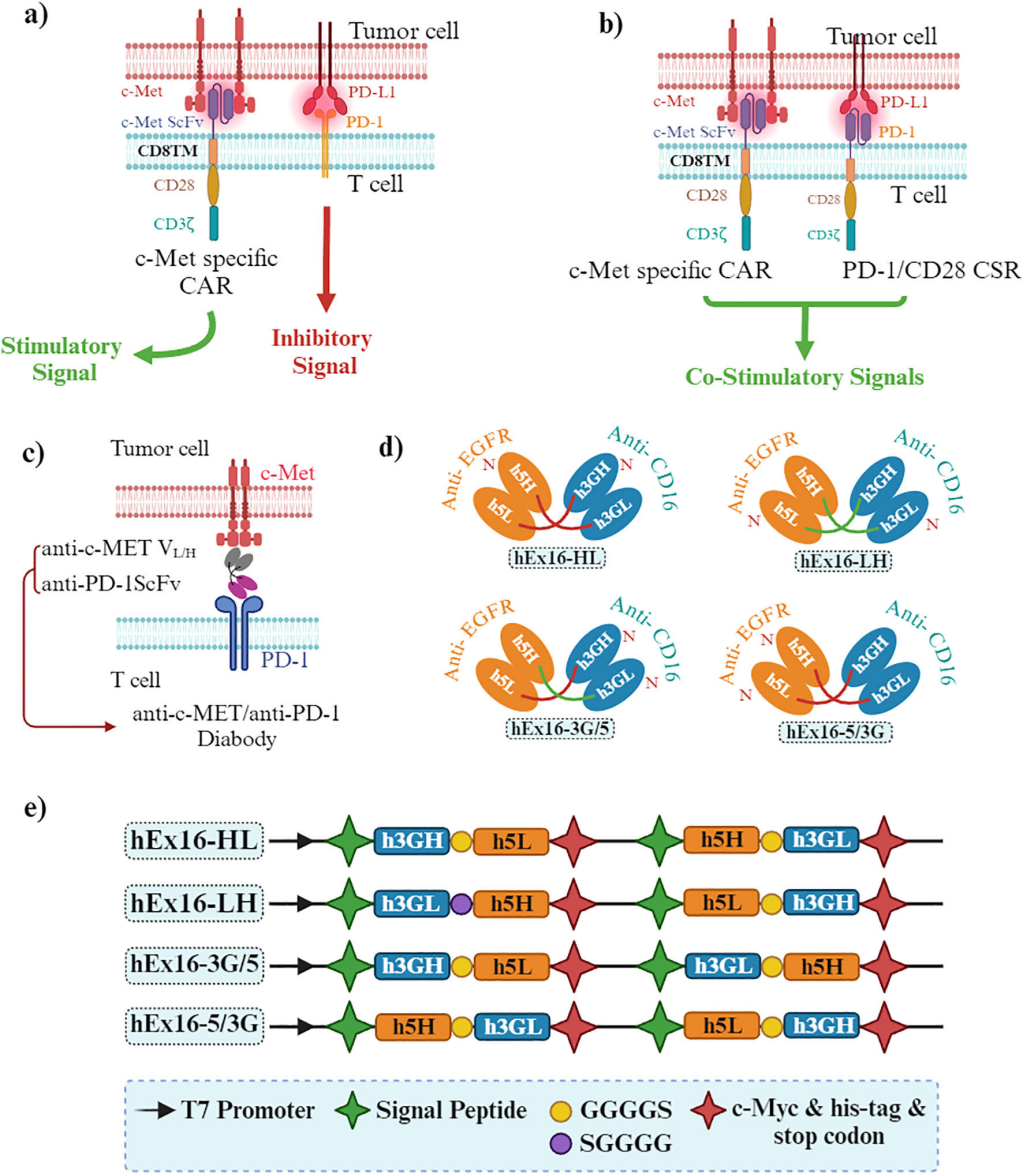
Bispecific therapeutic approaches against c-MET. **(A, B)** Innovative Therapeutic Tools for Dual PD-L1^+^ and c-MET^+^ Tumor Cells. **(A)** The interaction between PD-1 and PD-L1 counteracts the activating signals of c-MET-specific CAR-T cells. **(B)** The Supportive Role of PD-1-chimeric Switch Receptors (CSR). To counteract the inhibitory effect of PD-L1 on CAR-T cells, c-MET-specific CAR-T cells expressing PD-1-CSR amplify the stimulatory signals in T cells. **(C)** Bispecific Diabody Designed to Target Both c-MET and PD-1. **(D)** Bispecific Diabodies Targeting EGFR and CD16. **(E)** Schematic Diagrams of the Vectors Constructed for the Two Variants of hEx16-scDbs. Despite both domain orders exhibiting similar cross-linking abilities, one arrangement showed superior cytotoxicity in growth inhibition assays. In the case of hEx16-Dbs, domain order may influence the agonistic activity of the anti-CD16 segment, a notion supported by cytokine production tests, which likely explains the enhanced efficacy of one particular hEx16-Db. The HL-type domain arrangement exhibited greater growth inhibitory effects than the LH-type (129). *Created in BioRender. Jabbarzadeh Kaboli, P. (2024)**https://BioRender.com/q16b038*.

Nonetheless, the potent immunosuppressive tumor microenvironment (TME) poses a challenge. CAR-T cells unleash IFN-γ secretion, leading to the expression of PD-L1 in tumor cells, culminating in compromised CAR efficacy (127, 128). PD-1/CD28 chimeric switch receptors (CSR) have emerged, featuring PD-1 extracellular and CD28 intracellular domains, effectively transforming inhibitory PD-1 signals into stimulatory CD28 signals. This ingenious approach seeks to bolster the efficacy of c-MET-CARs, employing a fusion of c-MET/CD28-CAR-T and PD-1/CD28-CSR. *In vitro* and *in vivo* investigations yield promising results, marked by the downregulation of PD-L1 and enhanced efficacy of c-MET-CARs. Additionally, c-MET-CAR-T cells are found to elevate CD3^+^ CD8^+^ T cells and CD62L^+^ CCR7^+^ memory T cells, further enhancing their therapeutic potential (**Figures 7A, B**) (130).

## 6 Anti-MET diabodies

Diabodies, smaller engineered fragments, comprise two connected scFvs and can be bispecific, allowing them to bind to two distinct antigens or epitopes. In contrast to antibodies, which are mainly monospecific and widely used across various applications, diabodies provide better tissue penetration and dual-targeting abilities, which are especially useful in targeted cancer treatments (**Figure 7C**). Previous research highlighted the impact of domain order on the function of humanized bispecific diabodies targeting EGFR on cancer cells and CD3 on T cells, noting potential steric hindrance (131, 132). Bispecific diabodies can have their domains arranged in four distinct ways. However, the impact of domain order on the cytotoxicity of bispecific diabodies that redirect immune cells to attack tumor cells had not been previously examined (132). New studies on humanized bispecific diabodies targeting EGFR and CD16 on NK cells (hEx16-Dbs) predicted minimal steric effects due to CD16’s lack of accessory molecules (129) (**Figures 7C–E**).

The development of single-chain bispecific diabodies targeting c-MET and PD-1 for solid tumors has yielded notable effects, particularly in suppressing HGF/c-MET signaling, migration, and invasion of c-MET^+^ lung cancer and HCC cell lines such as A549 and MHCC-97H, respectively. Simultaneously, by blocking PD-1, these diabodies also direct T cells toward c-MET^high^ tumor cells. These diabodies, which target both c-MET^+^ tumor cells and PD-1^+^ immune cells, demonstrate efficacy across cell lines expressing varying levels of c-MET. The efficacy of c-MET/PD-1 diabodies is more significant than c-MET and PD-1 monotherapies such as capmatinib (an anti-c-MET monotherapy), toripalimab (an anti-PD-1 monotherapy), and the combination of capmatinib and toripalimab (17).

## 7 Conclusion and future direction

To understand the role of c-MET in cancer therapy, it’s crucial to recognize its multifaceted impact on targeted therapy and immunotherapy. Several studies have spotlighted how c-MET overexpression and hyperactivity correlate with PD-L1 expression, facilitating cancer cells’ evasion of the immune system’s anticancer defenses (12, 133). Conversely, c-MET activation promotes the transition of TAMs from the M1 to M2 phenotype and elevates immunosuppressive T cells in the TME (51). This intricate interplay underscores c-MET’s direct link to compromised treatment responses, including immunotherapy.

A review of single-cell analysis studies underscores c-MET’s pivotal role as a tumorigenic and metastatic driver, evident in the immunosuppressive TME and blood plasma (134, 135). However, amidst these challenges, a beacon of promise emerges as the bispecific c-MET/EGFR antibody, amivantamab. This innovative therapy has amplified the immune system’s antitumor efficacy through an Fc-dependent mechanism. Amivantamab’s Fc domain engages Fc gamma receptor (FcγR)-IIIa/CD16a on immune cells (136), triggering Fcγ activation in NK cells, monocytes, and macrophages. This, in turn, instigates ADCC, cytokine production, and antibody-dependent cellular trogocytosis (ADCT) (137). Significantly, this study reveals that monoclonal antibodies targeting RTKs like c-MET and EGFR possess dual functionality: they primarily inhibit RTKs and, in parallel, activate humoral and innate antitumor immunity (136). Amivantamab has garnered U.S. FDA approval for adult patients with locally advanced or metastatic NSCLC harboring EGFR Exon 20 insertion mutations (138). Encouragingly, a phase I clinical trial is underway, investigating the combination of amivantamab with an iPSC-derived CAR-NK cell product candidate (FT536) (NCT05395052).

The potential of c-MET as a therapeutic target takes center stage, offering the tantalizing prospect of dual inhibition against tumorigenesis and immunosuppression with a single c-MET inhibitor. Moreover, given c-MET’s diverse tumorigenic functions and its pivotal role in the invasiveness of aggressive tumors like NSCLC, the advent of c-MET-specific CAR-immune cells kindles hope for treating patients grappling with malignant tumors through more precisely tailored immunotherapeutic strategies targeting the formidable c-MET receptor. However, clinical trials underscore the need for personalized approaches, contingent on cancer type and phosphorylated c-MET status and emphasize that the role of c-MET-targeted therapy in immune cells warrants further exploration.

Nonetheless, there are inherent limitations in the immunotherapeutic targeting of c-MET. Activation of c-MET has been implicated in conferring resistance to immunotherapies, remarkably immune checkpoint blockade, by upregulating PD-L1, which dampens T cell activity. Consequently, solely targeting c-MET may not suffice to overcome this resistance. Additionally, clinical trials combining c-MET inhibitors with immunotherapies have yielded variable outcomes dependent on factors such as cancer subtype and patient demographics. This suggests that the efficacy of c-MET inhibition alongside immunotherapy might differ depending on the cancer type. Unfortunately, some clinical trials targeting c-MET with immunotherapy faced premature termination due to low enrollment rates, underscoring the challenges in conducting such trials and obtaining conclusive results. Furthermore, c-MET inhibition can affect various immune components within the tumor microenvironment, including TANs, TAMs, and Tregs. While these effects hold therapeutic potential, they also underscore the intricate web of interactions between c-MET and the immune system.

In conclusion, the intricate interplay between c-MET, targeted therapy, and immunotherapy offers promise in cancer treatment. However, it also presents a complex landscape with resistance mechanisms, variable clinical outcomes, and intricate immune interactions. Navigating these complexities is imperative in pursuing effective c-MET-targeted immunotherapies for cancer patients.
