# Correction: c-MET and the immunological landscape of cancer: novel therapeutic strategies for enhanced anti-tumor immunity

**DOI:** 10.3389/fimmu.2025.1729628

**Published:** 2025-11-06

**Authors:** Parham Jabbarzadeh Kaboli, Ghazaal Roozitalab, Reyhaneh Farghadani, Zoya Eskandarian, Abdessamad Zerrouqi

**Affiliations:** 1 Department of Biochemistry, Faculty of Medicine, Medical University of Warsaw, Warsaw, Poland; 2 Noncommunicable Diseases Research Center, Fasa University of Medical Sciences, Fasa, Iran; 3 Jeffrey Cheah School of Medicine and Health Sciences, Monash University Malaysia, Subang Jaya, Selangor Darul Ehsan, Malaysia; 4 Research Institute Children’s Cancer Center, and Department of Pediatric Hematology and Oncology, University Medical Center Hamburg-Eppendorf, Hamburg, Germany

**Keywords:** c-MET, immunotherapy, PD-L1, cancer, galectin, CAR-T, antibody-drug conjugate

An incorrect number was provided for “National Science Centre, Poland”. The correct number is “2020/39/B/NZ6/03513”.

In the published article, there was a mistake in the funding grant number. The funding grant number was displayed as “2020/39/NZ6/03513”. The correct grant number is “2020/39/B/NZ6/03513’’.

